# Regulation of Cell–Nanoparticle Interactions through Mechanobiology

**DOI:** 10.1021/acs.nanolett.4c04290

**Published:** 2025-01-08

**Authors:** Marco Cassani, Francesco Niro, Soraia Fernandes, Daniel Pereira-Sousa, Sofia Faes Morazzo, Helena Durikova, Tianzheng Wang, Lara González-Cabaleiro, Jan Vrbsky, Jorge Oliver-De La Cruz, Simon Klimovic, Jan Pribyl, Tomas Loja, Petr Skladal, Frank Caruso, Giancarlo Forte

**Affiliations:** a International Clinical Research Center, St. Anne’s University Hospital, 65691 Brno, Czech Republic; b Department of Chemical Engineering, The University of Melbourne, Parkville, Victoria 3010, Australia; c School of Cardiovascular and Metabolic Medicine & Sciences, King’s College London, London WC2R 2LS, U.K.; d Faculty of Medicine, Department of Biomedical Sciences, Masaryk University, 62500 Brno, Czech Republic; e Departamento de Química Física, Universidade de Vigo, Campus Universitario As Lagoas Marcosende, Vigo 36310, Spain; f Institute for Bioengineering of Catalonia (IBEC), The Barcelona Institute for Science and Technology (BIST), 08028 Barcelona, Spain; g Nanobiotechnology Core Facility, CEITEC Masaryk University, 62500 Brno, Czech Republic; h Department of Biochemistry, Faculty of Science, Masaryk University, 62500 Brno, Czech Republic; i Molecular Medicine, CEITEC Masaryk University, 62500 Brno, Czech Republic

**Keywords:** nanoparticles, bio−nano interactions, mechanobiology, mechanotransduction

## Abstract

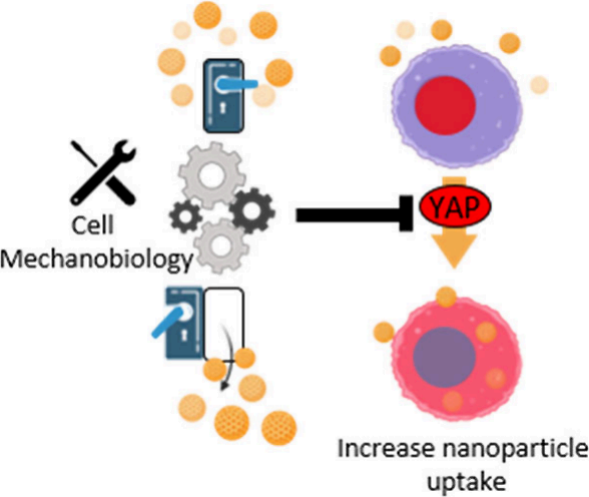

Bio–nano interactions have been extensively explored in nanomedicine to develop selective delivery strategies and reduce systemic toxicity. To enhance the delivery of nanocarriers to cancer cells and improve the therapeutic efficiency, different nanomaterials have been developed. However, the limited clinical translation of nanoparticle-based therapies, largely due to issues associated with poor targeting, requires a deeper understanding of the biological phenomena underlying cell–nanoparticle interactions. In this context, we investigate the molecular and cellular mechanobiology parameters that control such interactions. We demonstrate that the pharmacological inhibition or the genetic ablation of the key mechanosensitive component of the Hippo pathway, i.e., yes-associated protein, enhances nanoparticle internalization by 1.5-fold. Importantly, this phenomenon occurs independently of nanoparticle properties, such as size, or cell properties such as surface area and stiffness. Our study reveals that the internalization of nanoparticles in target cells can be controlled by modulating cell mechanosensing pathways, potentially enhancing nanotherapy specificity.

In recent decades, understanding the interactions between nanomaterials and biological systems has become a primary goal in nanomedicine, aiming to design nanomaterials (including nanoparticles, NPs) that can efficiently engage with living cells.^1−4^ While efforts have mostly focused on engineering the physicochemical properties of nanoparticles, such as size, shape, stiffness, and surface chemistry, with the goal to enhance their interaction with cells and improve drug delivery, the role of intracellular molecular pathways in bio–nano interactions has often been overlooked.^5−7^ Despite the significant progress achieved over the past two decades in understanding bio–nano interactions, there is a need to further elucidate mechanisms governing nanoparticle interactions with biological environments.^8,9^ Unveiling the processes responsible for nanomaterial–cell interactions at the molecular level may shed new light on nanoparticle transport within specific cells.^10^ In this context, cell mechanics has emerged as a promising area of investigation, revealing its role in regulating cell–nanoparticle interactions.^11−14^

Recently, mechanotherapeutics has emerged as a new class of drugs and treatments targeting mechanically activated pathways involved in various pathologies.^15^ Targeting mechanosensing pathways has resulted in promising outcomes in guiding cell fate and modulating cell function.^16^ The components of such pathways are responsible for controlling the expression of genes related to cell migration and survival, and cancer malignant progression through the recruitment of specific transcription factors.^17,18^ Among the key players in cell mechanosensing, yes-associated protein (YAP) has emerged as a central regulator of mechanotransduction in cancer cells.^19^ YAP, a mechanoactivated protein acting as the downstream effector of the Hippo pathway, is frequently dysregulated in cancer and contributes to cell proliferation, migration, survival, and immune evasion.^20−22^ We reported that YAP deletion in cancer cells leads to significant changes in cell shape and morphology, substrate adhesion, and the perception of mechanical cues generated within the surrounding microenvironment.^23^ More recently, our work revealed that YAP regulates cell–nanoparticle interactions and the delivery of nanodrugs by influencing cellular mechanical properties, the expression of genes involved in endocytic pathways, and the deposition of ECM components.^24^ Specifically, we demonstrated that the genetic or pharmacological inhibition of YAP significantly enhances nanoparticle internalization in the triple-negative breast cancer (TNBC) cell line CAL51, which is characterized by high YAP expression and transcriptional activity.^23^ To deepen our understanding of this phenomenon, in the present study, we investigated the role of YAP on nanoparticle uptake in a different cell model, i.e., HEK 293T, which displays a lower YAP expression and transcriptional activity than CAL51 (Figure S1).^24^ Despite HEK 293T cells showing lower dependence on YAP activity in terms of mechanical properties such as adhesion, shape, and stiffness, our findings further elucidate the role of YAP in cell–nanoparticle interactions.

Herein, by minimizing the influence of other factors, such as membrane stiffness and cell surface area, which may significantly influence cell–nanoparticle interactions, we sought to determine the unique role of YAP in this process. Additionally, our research highlights the potential for reducing YAP expression to enhance nanoparticle uptake. Thus, by modulating the activity of YAP, we sought to determine the role of the Hippo effector and its associated mechanoregulated pathways in directing the outcome of the nanoparticle association with cells.

Using CRISPR/Cas9 technology, we generated a stable YAP-deficient mutant HEK 293T cell line^24^ and confirmed YAP depletion through Western blot analysis (Figure 1a). At the transcriptional level, reverse transcription-quantitative polymerase chain reaction (RT-qPCR) showed a marked reduction in the mRNA levels of YAP accompanied by a decrease in the expression of connective tissue growth factor (CTGF), one of the main transcriptional targets of YAP (Figure 1b). The physical and mechanical properties of the HEK 293T cells remained unaltered after YAP depletion as determined by atomic force microscopy (AFM) (Figure 1c). Likewise, the cell surface area, as assessed by membrane extension and actin coverage, was unaffected (Figure 1d,e). Furthermore, confocal images revealed that wild-type (WT) HEK and YAP −/– HEK share the same morphology and membrane volume (Figure 1f,g and Figure S2). Noteworthy, the depletion of YAP in HEK 293 T cells has no effect on the cell’s proliferation and migration ability, as confirmed by proliferation assessment and wound healing assay (Figure S3). In addition, the adherence of HEK WT and YAP −/– cells to surface was evaluated by measuring their contact angle, revealing no difference in the way HEK 293 T cells grow attached to the substrate in the presence or absence of YAP (Figure S4).

**Figure 1:**
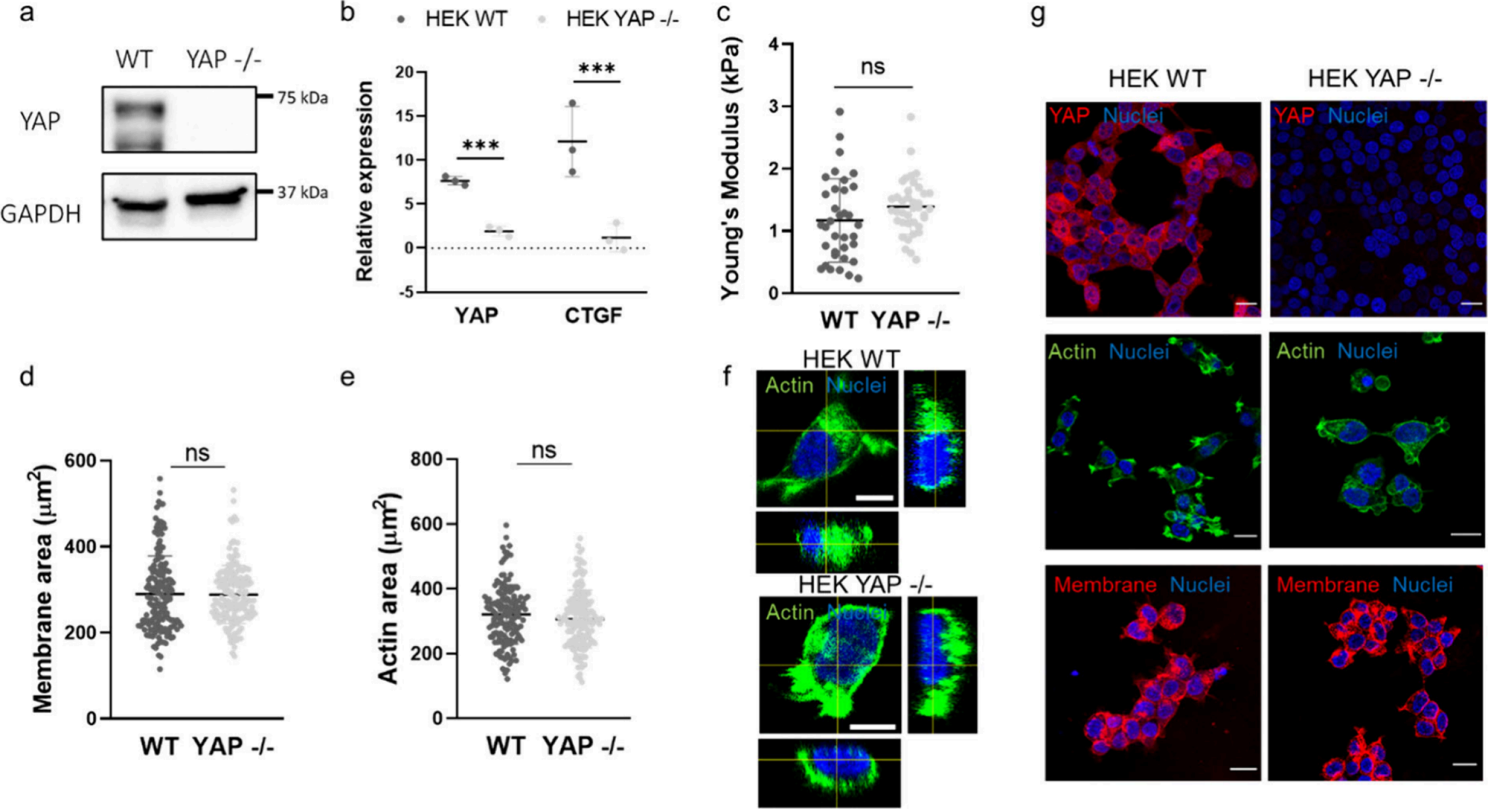
YAP depletion does not influence the adhesion, mechanics, or morphology properties of HEK cells. (a) The levels of YAP protein in WT and YAP −/– HEK cells as analyzed via Western blot. For protein loading normalization, β-tubulin was used. (b) RT-qPCR analysis of YAP and CTGF in WT and YAP −/– HEK cells. Statistical analysis was performed using multiple *t*-test; *n* = 3; ***p < 0.001. (c) Dot plots of the Young’s moduli of WT and YAP −/– HEK cells, as measured by AFM. Statistical analysis was performed using an unpaired *t*-test with Welch’s correction; ns, nonsignificant. (d) Dot plot of the total membrane area of WT and YAP −/– HEK cells. Alexa Fluor 488-labeled wheat germ agglutinin (WGA-488) was used to stain the cells; *n* > 100. Statistical analysis was performed using an unpaired *t*-test with Welch’s correction; ns, nonsignificant. (e) Dot plot analysis of the surface area of WT and YAP −/– HEK cells, as calculated on the basis of the total actin coverage of the cells. Alexa Fluor 488-labeled phalloidin (Pha-488, green) was used to stain the cells; *n* > 100. Statistical analysis was done using an unpaired *t*-test with Welch’s correction; ns, nonsignificant. (f) Three-dimensional (3D) reconstruction of WT and YAP −/– HEK cells. Cells were stained with 4′,6′-diamidino-2-phenylindole (DAPI) and Pha-488. Scale bars: 10 μm. (g) Representative confocal images depicting YAP expression in WT and YAP −/– HEK cells. Cells were stained for YAP (Alexa Fluor 555, red, top), actin (Pha-488, green, middle), membrane (wheat germ agglutinin–Alexa Fluor 647 conjugate (WGA-647), red, bottom) and nuclei were counterstained with DAPI. Scale bars: 20 μm.

These results indicate that YAP depletion in HEK 293T cells influences the expression of target genes and the YAP-related transcriptional activity of the cells but has minimal effects on the physical and mechanical properties of the cells.

As shown in our previous work,^24^ YAP activity hampered the internalization of nanoparticles in TNBC cell line CAL51. However, while the regulation of this process in CAL51 could be attributed to the significant effects that YAP displayed on cell morphology, adhesion, membrane structure, and ECM deposition, this may differ in HEK 293T as these effects were only marginal (see Figure 1).

To examine how this process is regulated in HEK cells, poly(methyl methacrylate) carboxylated spherical nanoparticles of 100 nm or carboxylated polystyrene (PS) spherical nanoparticles of 200 and 900 nm in diameter (denoted as PS100, PS200 and PS900, respectively; Figure S5) that were labeled with carboxytetramethylrhodamine or Alexa Fluor 488 were used to treat WT and YAP −/– HEK cells (incubation period of 4 h), and cell–nanoparticle interactions were investigated using confocal microscopy and flow cytometry. To be noted, no significant changes in particle size were observed upon functionalization with the fluorophores, while a slight change in surface charge indirectly confirmed the binding of the fluorescent molecules on their surface (Figure S6). The confocal analysis revealed that the PS nanoparticles preferentially bound to YAP −/– HEK cells rather than to WT cells (Figure 2a–c). This effect was independent of the nanoparticle size and was confirmed by flow cytometry, which showed nanoparticle uptake ratios in YAP −/– HEK higher than those in WT cells (Figure 2d and Figure S7). Importantly, no change in membrane stiffness was observed after nanoparticle binding, indicating that the cell–nanoparticle interactions did not significantly influence the mechanical properties of the cells (Figure 2e,f). To confirm these results, a different nanoformulation constituted of doxorubicin-loaded liposomes (Doxo-NPs) was used (Figure S8). These particles allowed us to test our findings on a different type of NP template, which is lipid-based compared to the polymer-based templates (i.e., poly(methyl methacrylate) and polystyrene) of the PS100, PS200, and PS900 particles. Additionally, the use of Doxo-NPs offers clinically relevant insights into the studied phenomenon, as liposomes have a long history of clinical application since the development of first FDA-approved nanodrug Doxil.^25^ Our findings confirmed higher NPs binding and internalization in HEK YAP −/– cells compared to WT cells (Figure S9). To further strengthen our findings, we also used 50 and 100 nm gold nanoparticles (AuNPs), widely used in bio–nano interaction studies,^26,27^ coated with a metal-phenolic network (MPN) using tannic acid and Co^II^ metal,^28^ and labeled with rhodamine B (Au@RhodB@MPN). The results revealed that decreased YAP expression correlates with increased Au@RhodB@MPN binding, confirming our previous observations (Figures S10 and S11).

**Figure 2:**
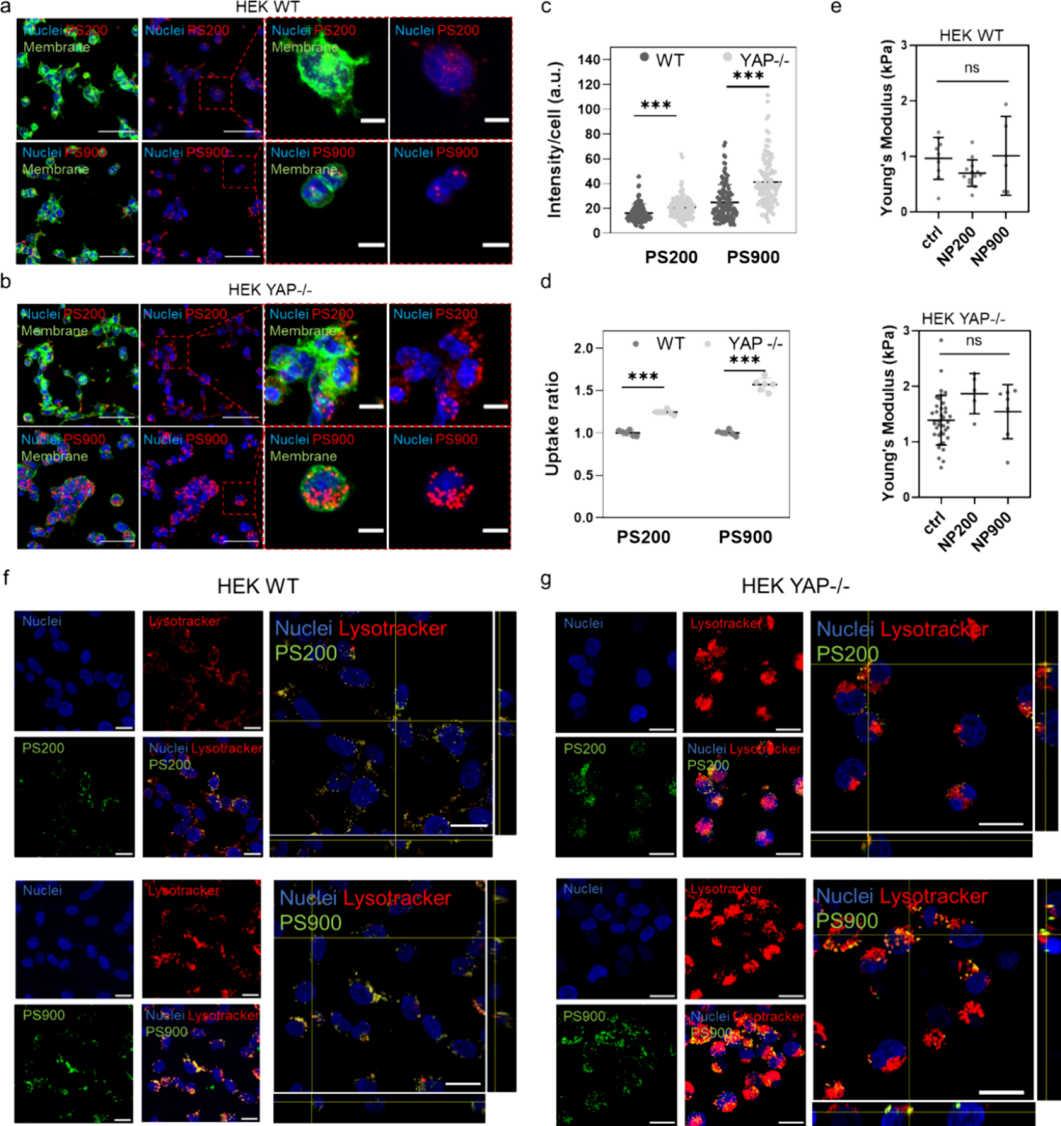
YAP regulates nanoparticle association with HEK 293T cells. (a, b) Representative confocal images of WT (a) and YAP −/– (b) HEK cells after incubation for 4 h with PS200 or PS900. Cells are stained with WGA-488 (green) and/or DAPI (blue). Magnified images of the regions within the red dashed boxes are also shown. Scale bars: 50 and 10 μm for the lower and higher magnification images, respectively. (c) Nanoparticle intensity per cell after incubation of PS200 or PS900 with WT and YAP −/– HEK cells for 4 h. Statistical analysis was performed using one-way analysis of variance (ANOVA) followed by Sidak’s multiple comparison test; *n* > 100; *p < 0.05; ***p < 0.001. (d) Uptake ratios of PS200 and PS900 in WT or YAP −/– HEK cells after incubation for 4 h. Statistical analysis was done using two-way ANOVA followed by Sidak’s multiple comparison test; *n* = 6; ***p < 0.001. (e, f) Young’s moduli of WT (e) and YAP −/– (f) HEK cells after incubation for 4 h with PS200 or PS900, as measured by AFM. Statistical analysis was performed using Kruskal–Wallis one-way ANOVA followed by Dunn’s multiple comparisons test; ns, nonsignificant. (f, g) Confocal images of the intracellular localization of PS200 (top) and PS900 (bottom) in HEK WT (f) and YAP −/– (g) cells after 4 h incubation with the nanoparticles. Cells are stained with DAPI (blue) and Lysotracker (red). Nanoparticles are displayed in green. Scale bar: 20 μm.

After binding to the cells, the internalization of the particles was followed using LysoTracker, which showed positive colocalization between all of the particles and the lysosomes (Figure S12 and Figures S13–S16). Furthermore, *z*-stack confocal images and imaging analysis performed via Imaris software confirmed the more effective internalization of the nanoparticles by YAP −/– cells, with a higher number of particles found to bind and colocalize with the cell membrane in the absence of YAP (Figures S17 and S18). As expected, this phenomenon was also observed at later time points (Figure S19). However, it is worth mentioning that the difference in cell binding between WT and YAP −/– cells appears to decrease over time. We propose that this observation may be attributed to the saturation of the internalization machinery within the cells, including the interaction between the cell membrane and particles, which tends to occur at extended incubation times. Data from the present manuscript (Figures 2f,g and S10–S17) and our previous study,^24^ indicate that while YAP depletion influences the dynamics of internalization, it does not affect the overall process of internalization itself. Compellingly, the same internalization trend was observed in cells seeded at both high and low confluency, with more particles found to interact with HEK YAP −/– compared to HEK WT cells (Figure S20).

Furthermore, we set out to investigate cell-NP interactions within 3D models by culturing spheroids of both WT and YAP −/– HEK cells. YAP depletion did not affect the overall size or morphology of the spheroids (Figure S21a–d). Interestingly, incubation with Doxo-NP led to significantly higher cell death in spheroids derived from HEK YAP–/– cells 72 h post-treatment (Figure S21e–g). This outcome suggests that nanoparticle penetration, and thus doxorubicin delivery, was more efficient in spheroids lacking YAP expression. These findings highlight potential therapeutic opportunities through the combination of mechanotherapy and nanomedicine.^4^

Collectively, our results show increased nanoparticle association and internalization in YAP −/– HEK cells despite the marginal effect that YAP depletion has on the physical and mechanical properties of HEK 293T cells.

To explore the processes responsible for the different interactions with nanoparticles, we conducted unbiased RNA sequencing (RNA-seq) analysis and evaluated changes induced by YAP depletion in the HEK 293T transcriptional landscape. The analysis revealed a total of 503 differentially expressed genes in YAP −/– HEK cells, with 297 of the expressed genes being downregulated and 206 of them being upregulated following YAP depletion (Figure S22). In virtue of the strong effect that YAP exert on extracellular matrix deposition and cell adhesion,^23^ the different regulation between HEK WT and YAP −/– cells of genes belonging to the human matrisome,^29,30^ and related to the gene ontology annotation associated with focal adhesion (GO:0005925) was evaluated. We observed upregulation of genes belonging to ECM-affiliated proteins, glycoproteins, collagens, secreted factors, and ECM regulator classes in both HEK WT and YAP −/– cells. However, only HEK WT cells exhibited upregulation of components of the proteoglycans class (Figure S23). Regarding focal adhesions, the most notable upregulated gene in HEK WT was vinculin (VCL) (Figure S24a). The upregulation of vinculin in HEK WT was confirmed via confocal analysis, which, however, revealed that the protein was unable to assemble into functional focal adhesions (Figure S24b). Notably, the most represented gene ontology (GO) annotations were associated with membrane organization, with 53% of genes downregulated in YAP −/– HEK involved in membrane organization (Figure 3a). Among the genes downregulated in YAP −/– HEK and involved in membrane organization, we identified *CAV1* (log_2_ fold-change (log_2_ FC) = 1.6), *RhoA* (log_2_ FC = 1.01), and *TLCD2* (log_2_ FC = 1.1) (Figure 3b,c and the Supporting Information RNaseq table).

**Figure 3:**
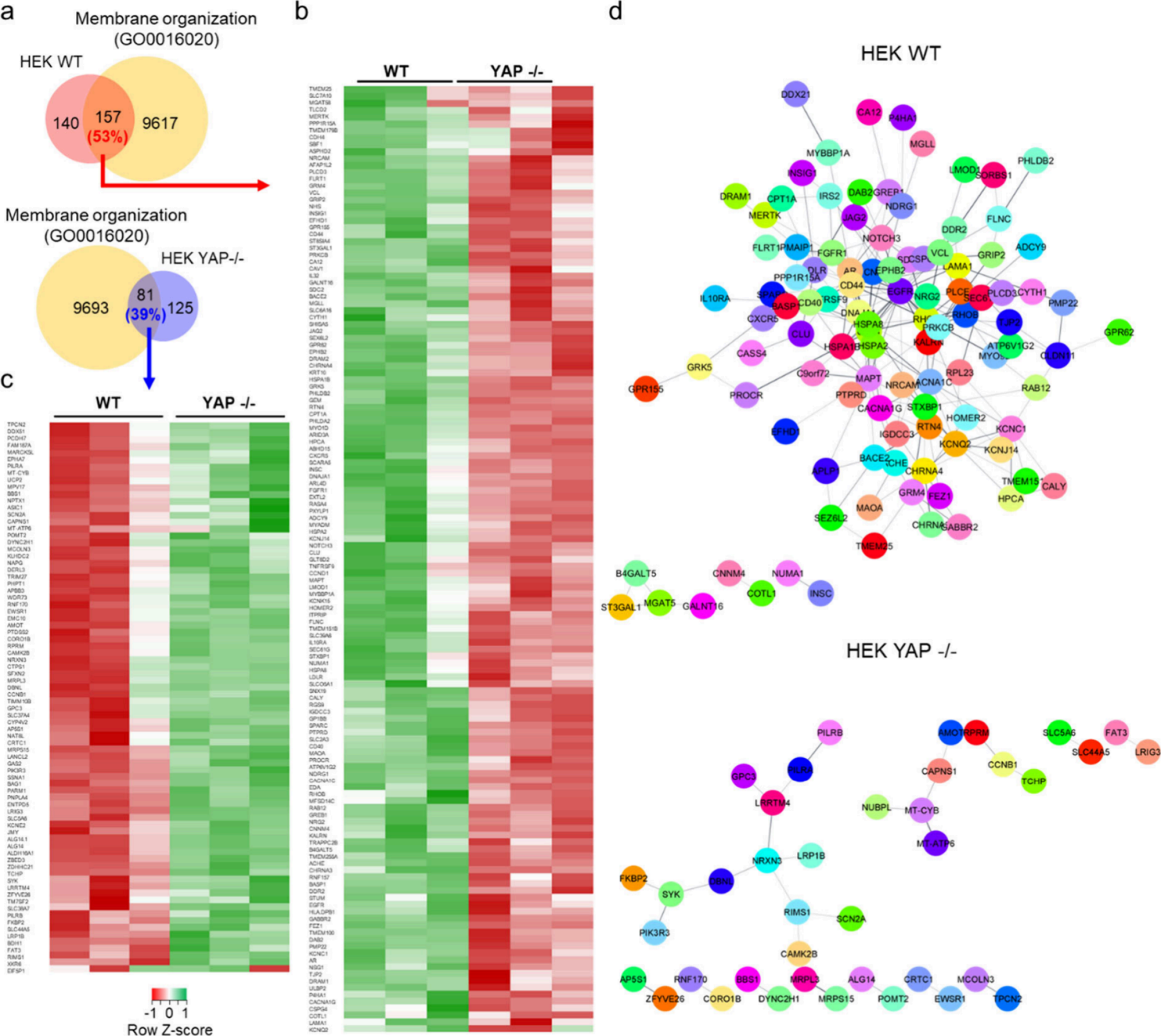
YAP depletion in HEK cells alters the expression of genes related to membrane organization. (a) Venn diagram showing the overlap between genes significantly downregulated in WT and YAP −/– HEK cells and belonging to the membrane organization network (GO0016020), as obtained by RNA-seq. (b, c) Heatmaps of the relative expression of representative differentially regulated genes associated with the membrane organization network, significantly downregulated (b) or upregulated (c) in YAP −/– HEK cells. *n* = 3 (*P*_adj_ <0.05, log_2_ FC > |1|). (d) STRING PPI network of differentially expressed proteins involved in membrane organization in WT and YAP −/– HEK cells obtained from Cytoscape (*P*_adj_ <0.05, log_2_ FC > |1|, confidence cutoff 0.4).

In addition, STRING PPI analysis yielded a highly clustered network (cluster coefficient 0.26) containing 15 nodes and 229 edges for the HEK WT. In contrast, only 81 nodes and 27 edges were identified in YAP-depleted cells (Figure 3d and Figure S25). WT HEK cells showed a higher number of interconnections than YAP −/– HEK cells, indicating that YAP regulates the expression of gene whose protein forms an intricated network in controlling membrane organization.

The cytosolic retention of YAP is commonly linked to protein turnover and degradation pathway alternative to the Hippo pathway, which involves large tumor suppressor homologue 1/2 (LATS1/2) phosphorylation and proteasomal degradation.^31^ However, recent research has unveiled the relationship between YAP and the cell membrane.^32,33^ These interactions may reveal novel and unexpected functions of the cytoplasmic pool of YAP in direct interaction with membrane proteins, vesicles, and organelles. Despite these possibilities, the precise role of YAP in these processes remains unclear.

Considering that the membrane organization could affect the exocytosis dynamics of the cells, once the NPs are internalized by the cells, we set to analyze this phenomenon by incubating HEK WT and YAP −/– cells with PS200 for 4 h and monitoring the retention of the particles at 24 and 48 h postincubation. Flow cytometry analysis revealed that despite a stark decrease of NPs retention for both WT and YAP–/– cells 24 h after incubation, HEK WT cells exhibit a further marked decrease at 48 h compared to YAP–/– cells (Figure S26). Although preliminary, these results indicate a distinct cell-NP interaction dynamic at the cell membrane level between WT and YAP-depleted cells. Furthermore, this finding may suggest differences in intracellular organelle dynamics, leading to increased NP retention in HEK YAP–/– cells. However, further experiments are required to understand this mechanism.

Collectively, the present findings suggest that interactions with nanoparticles are promoted in YAP-depleted cells owing to the dysregulation of an interconnected network of key regulators of membrane organization.

Given that HEK 293T cells displayed low cytoplasmic YAP expression that could not explain the differences in nanoparticle association found in CAL51 cell line,^24^ we conducted a series of experiments involving HEK 293T cells in which we induced increased YAP expression in the WT or knockout (KO) background. Cells were transfected with a plasmid carrying a transcriptionally hyperactive form of YAP, known as YAP S6A. This protein variant contains specific mutations that convert serine residues S61, S109, S127, S128, S131, S136, S164, and S381 into alanine residues.^34^ The accumulation of YAP in the cell nuclei is mostly controlled by a cascade of kinases of the Hippo pathway that phosphorylate YAP on serine residues, promoting its degradation in the cytosol and limiting its cotranscriptional activity (Figure 4a).^31^ The mutations carried by YAP S6A render the protein nonphosphorylatable and resistant to inactivation and degradation, resulting in the activation and translocation of the protein into the nucleus, where it can function as a transcriptional coactivator (Figure 4b).

**Figure 4:**
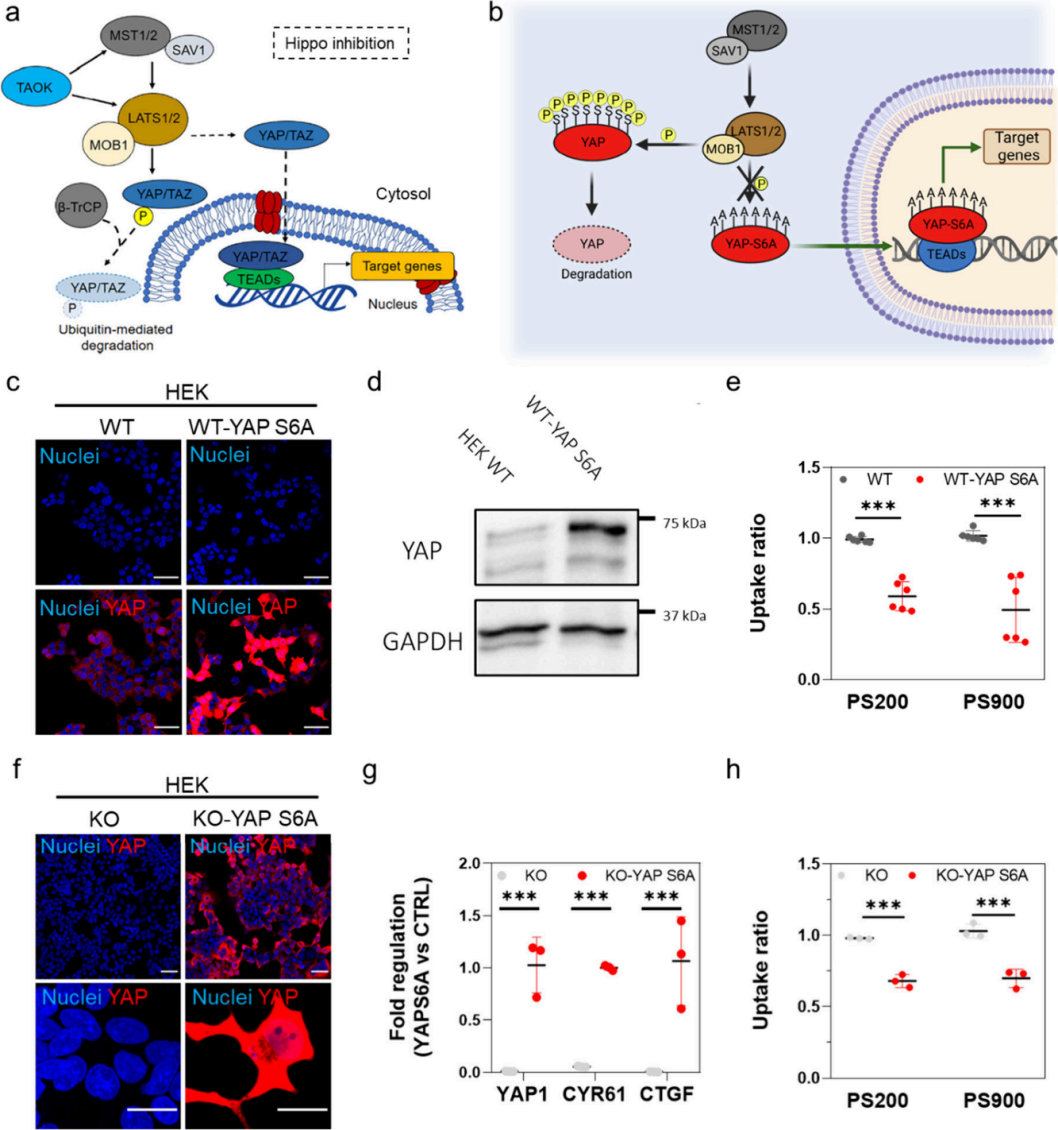
YAP overexpression or restoration in HEK 293T cells decreases their association with nanoparticles. (a) In condition of Hippo pathway activation, MST1/2 (STE20-like protein kinase 1/2) and SAV1 (protein salvador homologue 1) complex activates LATS1/2 (large tumor suppressor homologue 1/2) that, in association with MOB1 (MOB kinase activator 1), phosphorylates YAP and promotes its degradation. Conversely, when the Hippo signaling is inactive, YAP shuttles into the nucleus where it binds to TEADs (TEA domain transcription factor family members) and regulates the transcription of genes involved in cell proliferation, migration, and survival.^31^ TAOK, serine/threonine-protein kinase TAO1; β-TrCP, β-transducin repeat-containing proteins; TAZ, transcriptional coactivator with PDZ-binding motif. (b) Schematic representation of the constitutively active translocation of mutant YAP S6A to the cell nucleus. Owing to the substitutions of serine residues with alanine residues in different positions (S61A, S109A, S127A, S128A, S131A, S136A, S164A, and S381A), YAP-S6A cannot be phosphorylated by upstream kinases, mainly belonging to the Hippo pathway (LATS1/2 kinases and scaffolding protein MOB1). Created with Biorender.com. (c) Representative confocal images of WT and WT HEK cells transfected with YAP S6A (WT-YAP S6A). Cells were decorated with DAPI (blue) and YAP (red). Scale bars: 50 μm. (d) Western blot analysis of the levels of YAP protein in WT HEK cells and WT-YAP S6A cells. Glyceraldehyde 3-phosphate dehydrogenase (GAPDH) was used for protein loading normalization. (e) Uptake ratios of PS200 and PS900 by WT HEK or WT-YAP S6A cells after incubation for 4 h. Statistical analysis was done using two-way ANOVA followed by Sidak’s multiple comparison test; *n* = 6; ***p < 0.001. (f) Confocal images of YAP −/– HEK (KO) cells and YAP −/– HEK cells transfected with YAP S6A (KO-YAP S6A). Scale bars: 50 and 10 μm in the lower and higher magnification images, respectively. (g) RT-qPCR analysis of YAP1, CYR61, and CTGF in KO and KO-YAP S6A cells. Statistical analysis was done using multiple *t*-test; *n* = 3; ***p < 0.001. (h) Uptake ratios of PS200 and PS900 in KO or KO-YAP S6A (red) cells. Statistical analysis was performed using two-way ANOVA followed by Sidak’s multiple comparison test; *n* = 3; ***p < 0.001.

YAP expression in transfected WT HEK cells was first confirmed by using confocal imaging (Figure 4c and Figure S27a) and Western blot analysis (Figure 4d). Subsequently, the transfected cells were incubated with PS200 or PS900 for 4 h. WT-YAP S6A HEK cells exhibited reduced nanoparticle uptake relative to the WT HEK control cells, as indicated by the uptake ratio obtained by flow cytometry (Figure 4e and Figure S27b,c). To further support these results, YAP −/– HEK cells were transfected with the plasmid carrying YAP S6A, and the expression of the protein was confirmed via confocal imaging (Figure 4f). We note that the reintroduction of YAP into the KO cells increased the mRNA levels of CYR61 and CTGF, two of the main transcriptional targets of YAP, as assessed by RT-qPCR (Figure 4g). Additionally, the reintroduction of YAP in YAP −/– HEK significantly decreased the uptake ratio of the nanoparticles by the cells (Figure 4h).

Together, these results corroborate the key role of YAP in regulating interactions between HEK cells and nanoparticles.

Given that YAP-induced expression leads to a reduction in nanoparticle association, herein, we sought to determine whether the pharmacological inhibition of the Hippo pathway kinase STE20-like protein kinase 1/2 (MST1/2) using the exogenous inhibitor XMU-MP1 could modulate cell interactions with nanoparticles.

XMU-MP1 is a small molecule inhibitor that blocks MST1/2 kinase, thereby preventing the activation of LATS1/2 and promoting YAP activation downstream of the Hippo pathway, leading to its nuclear shuttling (Figure 5a).^35^ To assess the effect of XMU-MP1 treatment on YAP expression, WT HEK cells were treated with 6 μM XMU-MP1 and the levels of the proteins downstream of the Hippo pathway were evaluated at different time points. Western blot analysis revealed a time-dependent decrease of MST1 (whereas the overall level of MST2 remained stable; Figure S28) and a decrease of pospho-MOB1 (p-MOB1, Figure 5b and Figure S28) in cells treated with XMU-MP1. This result indicates that treatment with XMU-MP1 is effective at reducing the activity of the target kinase. Importantly, this treatment did not induce significant toxicity in WT HEK cells, as confirmed by a live/dead assay (Figure S29).

**Figure 5:**
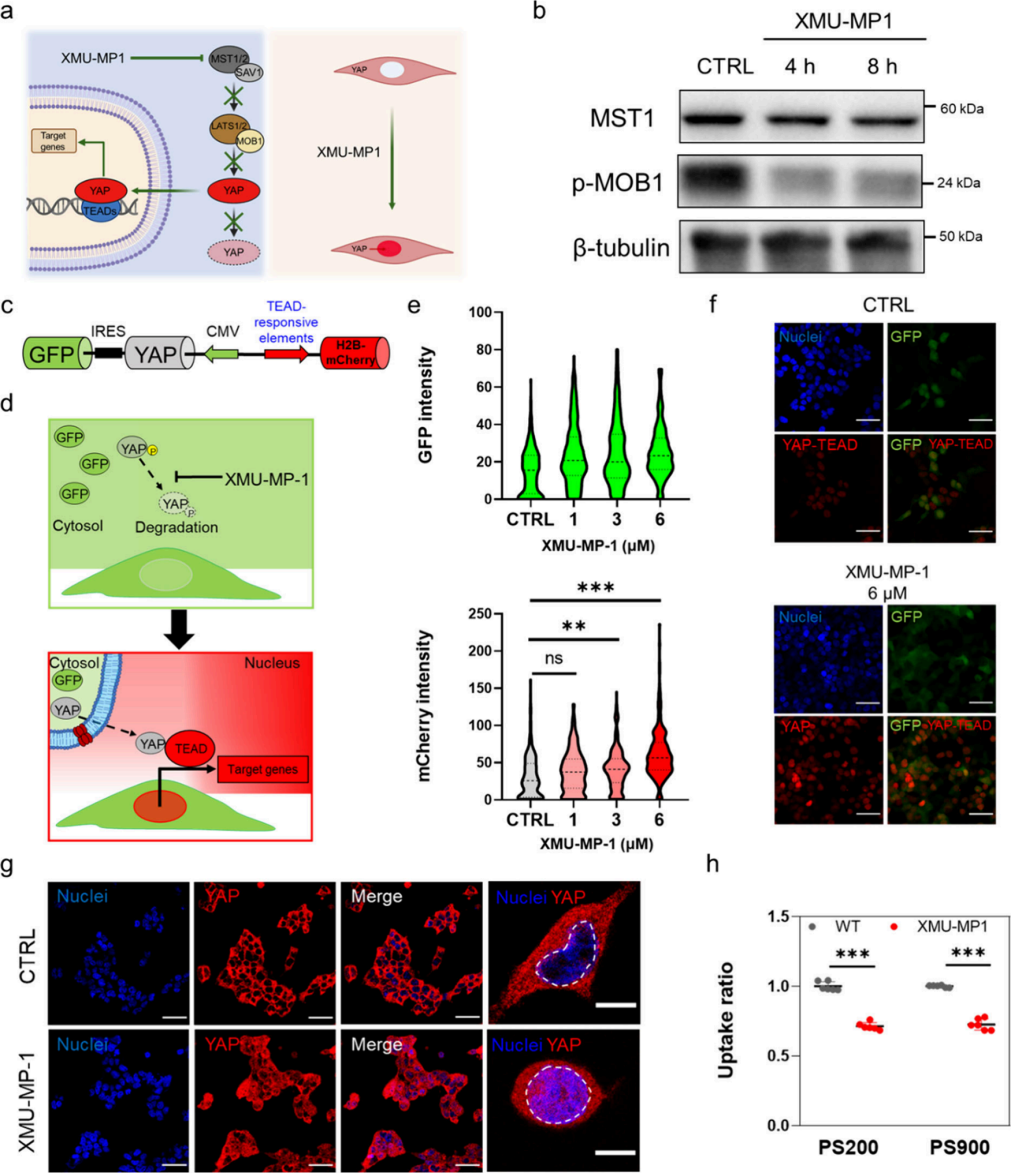
Suppression of the Hippo pathway with MST1/2 inhibitor XMU-MP1 in HEK 293T cells increases YAP activity and reduces cell–nanoparticle interactions. (a) Schematic representation of the effect of XMU-MP1 treatment. The drug inhibits the Hippo pathway by blocking the activity of upstream kinase MST1/2, thus suppressing the degradation of YAP and promoting its shuttling into the nucleus. Created with Biorender.com (b) Western blot showing the levels of MST1 and p-MOB1 in untreated HEK 293T cells (CTRL) or HEK 293T cells treated for 4 or 8 h with the 6 μM XMU-MP1 inhibitor. GAPDH was used for protein loading normalization. (c) Scheme of the reporter construct used in this study, as described by Maruyama et al.^36^ FLAG-His 6-YAP1 (FH-YAP1) gene is followed by IRESs and GFP gene is cloned under a CMV promoter. Histone 2B-mCherry (H2B-mCherry) gene is regulated under the TEAD-responsive element. (d) Schematic showing that treatment with XMU-MP1 increases the H2B-mCherry signal, as the YAP-mediated TEAD transcriptional activity is promoted owing to inhibition of the activity of upstream kinase MST1/2 of the Hippo pathway. (e) (Top) Violin plot of the GFP signal intensity in HEK 293T cells treated with increasing concentrations of XMU-MP1 for 8 h. (Bottom) Violin plot of the signal intensity of H2B-mCherry in HEK 293T cells treated with increasing concentrations of XMU-MP1 for 8 h. Statistical analysis was performed using one-way ANOVA followed by Tukey’s multiple comparison test; *n* > 200 cells; ns, nonsignificant; **p < 0.01; ***p < 0.001. (f) Representative confocal images of untreated WT HEK cells (CTRL) and WT HEK cells treated with 6 μM XMU-MP1 for 8 h. The green signal comes from GFP coexpressed with YAP, whereas the red signal comes from the YAP-TEAD-mediated gene transcription (mCherry). Cells are stained postfixation with DAPI (blue). Scale bars: 100 μm. (g) Representative confocal images of untreated HEK 293T cells (CTRL) and HEK 293T cells treated with 6 μM XMU-MP1 for 8 h. Cells are stained with DAPI (blue) and for YAP (Alexa Fluor 555, red). The magnified images show single untreated and treated cells with the nuclei delimited by the white dashed lines. Scale bars: 100 and 10 μm for the low and high magnification images, respectively. (h) Uptake ratios of PS200 and PS900 in untreated HEK 293T cells (CTRL) or HEK 293T cells treated with 6 μM XMU-MP1 for 8 h and incubated with the particles for 4 h (after 4 h of treatment with the inhibitor). Statistical analysis was performed using two-way ANOVA followed by Sidak’s multiple comparison test; *n* = 6; ***p < 0.001.

Studies were then conducted to gain spatiotemporal insights into YAP activation following XMU-MP1 treatment and to evaluate the potential to finely modulate YAP activity, and consequently cell–nanoparticle interactions, using this inhibitor. WT HEK cells were transfected with a YAP transcriptional reporter constituted by mCherry-fused histone2B (H2B-mCherry) under the TEAD-responsive promoter and FLAG-tagged YAP1 linked to green fluorescent protein (GFP) via the internal ribosome entry site (IRES) under the cytomegalovirus promoter (CMV), as previously reported (Figure 5c).^36^ Upon transfection with the reporter, YAP levels could be detected through the green signal resulting from GFP coexpression. In contrast, the transcriptional activity of YAP was identified by the red signal, which arises from YAP translocating to the nucleus and transcribing the YAP-TEAD-mediated reporter (as depicted in Figure 5d).

After transfection with the reporter, WT HEK cells were sorted and incubated with XMU-MP1. A stable green signal was observed from the GFP in the transfected cells, and treatment with the inhibitor for 8 h determined a concentration-dependent increase of mCherry signal (Figure 5e). This result indicates that treatment with XMU-MP1 effectively activates YAP-TEAD transcriptional activity in the cells owing to its inhibitory activity on MST1/2 kinase (as demonstrated by Western blot in Figure 5b) and consequent YAP nuclear shuttling and transcriptional activity upon interaction with TEAD (Figure 5f and Figure S30). Treatment of HEK 293T cells at the highest concentration of XMU-MP1 studied (i.e., 6 μM) for 8 h resulted in the translocation of YAP into the nucleus, as confirmed by confocal analysis (Figure 5g and Figure S31a–d). Noteworthy, flow cytometry analysis showed a decrease in cell–nanoparticle association after incubation for 4 h with the inhibitor for both PS200 and PS900 (Figure 5h and Figure S31e,f). Despite the overall level of YAP remaining constant, while the level of p-YAP varied (Figure S32), our functional activity assay and the protein colocalization study demonstrated a significant increase in YAP activity upon XMU-MP1 treatment.

Collectively, the findings demonstrate a strong correlation between the modulation of YAP activity and the association of nanoparticles with the cells.

In conclusion, we demonstrated that depleting YAP in HEK 293T cells led to an increase in nanoparticle uptake, independent of nanoparticle size, thus underscoring the critical role of YAP activity in this process. This phenomenon highlights a potential mechanotargeting effect, where the cell mechanical response plays a prominent role in processing bio–nano interactions. Substrate properties and cell mechanics are often closely interconnected and have been shown to influence bio–nano interactions and nanoparticle uptake processes.^37^

Through RNA-seq analysis, we showed that YAP was involved in the transcription of genes related to membrane organization. WT HEK cells exhibited an extensive network of components at the cell membrane level, whereas YAP −/– HEK cells displayed a looser network, indicating a lower level of membrane interconnections. Building on these findings and our previous data,^24^ which also showed a less interconnected network of membrane protein in YAP-depleted CAL51 cells, we hypothesize that the level of membrane organization in cells, which is highly dependent on YAP activity, impacts cell–nanoparticle association, thus shedding light on the potential impact of mechanobiology in shaping the dynamics of bio–nano interactions. While our results indicate the transcriptional role of the protein as the primary factor responsible for this phenomenon, the significantly lower YAP level in HEK cells compared to CAL51, and its relatively higher cytoplasmic localization in the former, may suggest that other mechanisms, influenced by the protein at different levels and potentially dependent on the cytosolic pool of YAP, could be involved in bio-nano interactions processes and warrant further investigation.

Although further research is needed to unravel the intricate interplay between mechanosensing and nanomaterials, our study offers a fundamental mechanism to explain nanoparticle internalization. A more thorough understanding of the cell mechanobiology pathways involved in bio–nano interactions, along with the identification of new targets and drugs to modulate these functions, could lead to the development of next-generation nanotherapies.

## Supporting Information Available

Table of the analysis of differential gene expression between HEK 293T WT vs HEK 293T YAP KO (XLSX)
Materials and methods, cell characterization, nanoparticles characterization, cell–nanoparticle interactions characterization, colocalization studies, RNaseq analysis, STRING analysis, Western blot quantification (PDF)
