# Supplementary material for: Regulation of Cell–Nanoparticle Interactions through Mechanobiology
Source: Nano Lett. 2025 Jan 8;25(7):2600–9. doi: 10.1021/acs.nanolett.4c04290 (PMC11849000; doi:10.1021/acs.nanolett.4c04290)
Supplement: Supplementary file 2 — nl4c04290_si_002.pdf [file nl4c04290_si_002.pdf]

Supporting information

Regulation of cell-nanoparticle interactions through mechanobiology

Marco Cassani,[a,b] Francesco Niro,[a,c,d] Soraia Fernandes,[a,b] Daniel Pereira-Sousa,[a,d] Sofia Faes Morazzo,[a,d] Helena Durikova,[a,b] Tianzheng Wang,[b] Lara González-Cabaleiro,[e] Jan Vrbsky,[a] Jorge Oliver-De La Cruz,[a,f] Simon Klimovic,[g,h] Jan Pribyl,[g] Tomas Loja,[i] Petr Skladal,[h] Frank Caruso*[*b] and Giancarlo Forte[*a,c]*

Affiliations

[a]International Clinical Research Center, St. Anne's University Hospital, 65691, Brno, Czech Republic.

[b]Department of Chemical Engineering, The University of Melbourne, Parkville, Victoria 3010, Australia.

[c]School of Cardiovascular and Metabolic Medicine & Sciences, King's College London, London WC2R 2LS, UK.

[d]Faculty of Medicine, Department of Biomedical Sciences, Masaryk University, 62500, Brno, Czech Republic.

[e]Departamento de Química Física Universidade de Vigo, Campus Universitario As Lagoas Marcosende, Vigo 36310, Spain.

[f]Institute for Bioengineering of Catalonia (IBEC), The Barcelona Institute for Science and Technology (BIST), 08028, Barcelona, Spain.

[g]Nanobiotechnology Core Facility, CEITEC Masaryk University, 62500, Brno, Czech Republic.

[h]Department of Biochemistry, Faculty of Science, Masaryk University, 62500, Brno, Czech Republic.

[i]Molecular Medicine, CEITEC Masaryk University, 62500, Brno, Czech Republic.

*Corresponding author. E-mail: mcassani@unimelb.edu.au (M.C.); fcaruso@unimelb.edu.au (F.C.); giancarlo.forte@kcl.ac.uk (G.F.)

This PDF file includes:

- Materials and Methods
- Figs. S1 to S32
- Reference S1-S10

MATERIALS AND METHODS

Materials. The following materials were used in the present study: Opti-Link carboxylate-modified particles (83000520100290 and W090CA, Thermo Fisher Scientific); PMMA carboxylate-modified 100 nm particles (PMMA-COOH-AR1132, Microparticles gmbh); Dox-NP (300112, Avanti Polar Lipids); 50 nm gold nanoparticles (753645, Merck); 100 nm gold NPs (742031, Merck) Dulbecco's modified Eagle medium (DMEM, Merck); TrypLE Express (12604013, Thermo Fisher Scientific); Opti-Mem™ medium (31985062, Thermo Fisher Scientific); 6-well plate (30006, SPL Life Sciences); penicillin/streptomycin (97063-708, VWR); 12-well plate (30012, SPL Life Sciences), 24-well plate (30024, SPL Life Sciences); μ-slide 8-well glass bottom dish (80807, Ibidi), tissue culture dish 40 mm (93040, iBiotech Ltd.); Pha-488 (A12379, Thermo Fisher Scientific); WGA-647 (W32466, Thermo Fisher Scientific); WGA-488 (W11261, Thermo Fisher Scientific); pLX304 (Addgene plasmid # 25890; http://n2t.net/addgene:25890; RRID:Addgene_25890); YAP1 (S6A) - V5 in pLX304 (Addgene plasmid # 42562; http://n2t.net/ addgene:42562; RRID:Addgene_42562); shYAP plasmid (sc-38637-SH, Santa Cruz Biotechnology); FuGENE™ HD transfection reagent (E2311, Promega); lipofectamine 3000 (L3000001, Thermo Fisher Scientific); 10% mini-protean TGX precast protein gel (4561033, Bio-Rad); protease and phosphatase inhibitor cocktails (PPC1010, Merck); RIPA buffer (R0278, Merck); horseradish peroxidase (HRP)-conjugated anti-rabbit and HRP-conjugated anti-mouse (RABHRP1 and RABHRP2, Merck); MOWIOL 4-88 reagent (475904, Merck); High Pure RNA Isolation Kit (11828665001, Roche); LIVE/DEAD viability/cytotoxicity Kit (L3224, Thermo Fisher Scientific); Sytox Blue Dead Cell Stain (S34857, Thermo Fisher Scientific); TAMRA cadaverine (92001, Biotium); Alexa Fluor 488 cadaverine (A30676, Thermo Fisher); rhodamine B (R6626, Merck); LysoTracker Deep Red (L12492, Thermo Fisher Scientific); LysoTracker green DND-26 (#8783, Cell Signalling); CellTrace CFSE Cell Proliferation Kit (C34554, Thermo Fisher Scientific); Carbon Support Film 5-6 nm on Square 200 mesh Cu Grid (Electron Microscopy Sciences CF200-Cu-50); Float-A-Lyzer G2 dialysis device (300 kDa cutoff; G235036, Merck); *N*-hydroxysuccinimide (804518, Merck); inhibitor XMU-MP1 (S8334, Selleckchem); *N*-(3-dimethylaminopropyl)-*N*′-ethylcarbodiimide (EDC)-hydrochloride (59002, VWR); 4′,6-diamidine-2′-phenylindole dihydrochloride (10236276001, Merck); mouse anti-YAP (4912, Cell Signaling); mouse anti-Vinculin (V9131, Merck); rabbit anti-YAP (14074, Cell Signaling Technology; 1:1000); mouse anti-β-tubulin (T8328, Merck); rabbit anti-MST1 (3682, Cell Signaling Technology); rabbit anti-MST2 (3952, Cell Signaling Technology); rabbit anti-p-YAP (Ser397) (D1EZY, 13619, Cell Signaling Technology); rabbit anti-p-MOB1 (Thr35) (8699, Cell Signaling Technology); mouse anti- Glyceraldehyde 3-phosphate dehydrogenase (GAPDH)-peroxidase (69295, Merck); anti-rabbit immunoglobulin G (IgG), HRP-linked antibody (7074P2, Cell Signaling Technology); and Precision Plus Protein Dual Color Standards (1610374, Bio-Rad, 3 μL/well). All antibodies used for western blotting were diluted at a ratio of 1:1000 or 1:500 unless otherwise specified. Antibodies for immunohistochemistry were diluted at a ratio of 1:200.

PS nanoparticle fluorescence labeling. Carboxylated PS particles with a diameter of 900 and 200 nm and carboxylated PMMA nanoparticles of 100 nm were labeled with TAMRA cadaverine or AF488 cadaverine, as previously reported.[S1] Briefly, the particles were resuspended in 5 mL 2-(N-morpholino)ethanesulfonic acid

(MES) buffer (50 mM, pH = 6.04) at a final concentration of 20 mg mL^{-1} and incubated with 52 μM *N*-(3-dimethylaminopropyl)-*N*′-ethylcarbodiimide hydrochloride and 5.2 μM *N*-hydroxysuccinimide for 1 h in an ice bath under magnetic stirring. TAMRA cadaverine or AF488 cadaverine was then added, and the mixture was brought to a final concentration of 50 μM and left to react overnight at room temperature. Following reaction, the particles were collected, centrifuged (5000 g for 10 min for the 900 nm particles and 12,000 g for 15 min for the 200 nm particles), and washed five times with distilled water. Subsequently, the particles were dialyzed against distilled water for 72 h using a Float-A-Lyzer dialysis device (300 kDa cutoff).

Metal-TA coating on AuNPs. Commercially available gold nanoparticles (AuNPs) of 50 nm (OD 1, stabilized suspension in 0.1 mM PBS); and 100 nm (OD 1, stabilized suspension in 0.1 mM PBS) in size were used for the coating. 500 µL of AuNPs were dispersed in 500 µL of milli-Q water, colloidally stabilized with 20 µL of SDS 25 % and codified with 20 µL of rhodamine B 0.1 mM, the AuNPs were incubated for 30 minutes undisturbed to allow the binding of the dye towards the surface of the AuNPs by electrostatic interactions. Afterwards, the AuNPs were washed by centrifugation (4000 g, 10 min) to remove the excess of the rhodamine B and redispersed in 500 µL of mili-Q water with 5 µL of Tween20 25%. Then 40 µL of TA solution (10 mg mL−1), 40 µL of Co(II) (2 mg mL-1) and 500 µL of MOPS, (100 mM, pH 8) to raise the pH and enable the complexation of the TA with Co (II) cations, were sequentially added under vortex stirring. After 1 hour of undisturbed incubation, the AuNPs were washed twice with milli-Q water by centrifugation (4000 g, 10 min) to remove excess of metal ion and TA. After each wash, the solution was sonicated for 30 s. Finally, Metal– phenolic network (MPN)-coated gold nanoparticles (Au@RhodB@MPN) were dispersed in 100 µL milli-Q water.

Generation of YAP mutant HEK 293T lines. The YAP −/− HEK 293T lines were generated using CRISPR/Cas9 technology as described previously.[2] Briefly, a guiding RNA was designed to target exon 1 of the YAP1 gene, which is common in all nine YAP1 splicing variants. Two sets of complementary single-stranded DNA oligonucleotides (YAP1_R1: 50-CACCgtgcacgatctgatgcc-30, YAP1_R2: 50-AAACccgggcatcagatcgtgcac-30, YAP1_F1: 50-CACCGcatcagatcgtgcacgt-30, YAP1_F2: 50-AAACcggacgtgcacgatctgatgC-30) were then cloned into pSpCas9(BB)-2A-GFP (PX458) and transfected into HEK 293T cells using the FuGENE HD transfection reagent according to the manufacturer's protocol. GFP-positive cells were sorted via fluorescence-activated cell sorting (MoFlo Astrios, Beckman Coulter, California, USA) as single cells and clonally propagated. Genomic DNA was sequenced from both sides to map the size of the deletion (sequencing primers: 50-gattggacccatcgtttgcg-30, 50-gtcaagggagttggagggaaa-30, 50-gaagaaggagtcgggcagctt-30, 50-gagtggacgactccagttcc-30).

Cell culture. WT HEK 293T (gifted by Dr. V. Pekarik, Department of Physiology, Masaryk University, Brno, Czech Republic) and mutant cell lines were cultured in DMEM containing 10% inactivated fetal bovine serum, 1% penicillin-streptomycin, and 1% glutamine at 37 °C, 95% humidity, and 5% CO_2. The cells were split every 2–3 days before reaching confluence, and only HEK 293T cells with a passage number of <10 were used for all experiments. Flow cytometry analysis was performed using unstained cells as a control. Cell viability was assessed using a LIVE/DEAD viability/cytotoxicity kit for mammalian cells, according to manufacturer's instructions. The cell proliferation assay was performed using the CellTrace CFSE Cell Proliferation Kit (C34554, Thermo Fisher). Cells were labeled with the dye according to the supplier's protocol and seeded onto a 24-well plate (30024, SPL Life Sciences) for 24, 48 and 72 hours. The analysis was performed using unstained cells as a

control. For wound healing assay, cells were seeded onto 12-well plate (30012, SPL Life Sciences) at 80% confluency. The day after, a scratch was generated in the center of each well using a 10 µL tip and wounding area assessed up to 56 hours. Image analysis was done with Image J, using “Wound healing size tool” plugin.

Spheroids from HEK cells were generated by seeding 10,000 cells per well in a volume of 100 µL in 96-well ultralow attachment plates and aggregating them via centrifugation at 500 g for 10 min. Alternatively, 100,000 cells per well in a volume of 500 µL were seeded in a 24-well ultralow attachment plate until the spheroids were formed. The spheroids were left to grow for 3 days with media replacement on day 2. The cell dead assay was performed using the Sytox Blue Dead Cell Stain (S34857, Thermo Fisher Scientific). Cells were labeled with the dye according to the supplier‟s protocol and seeded onto a 24-well plate (30024, SPL Life Sciences) for 24 hours. Flow cytometry analysis was performed using unstained cells as a control.

AFM measurements. Cells were seeded onto a 40 mm tissue culture (TPP Techno Plastic Products, Trasadingen, Switzerland) at a concentration of 100,000 cells per dish for 24 h. Force maps were measured using a bio-AFM Nanowizard 4XP (Bruker-JPK, Germany) placed on a Leica DMi 8 inverted microscope with a 10× objective (Leica, Germany). A 5.73 µm melamine sphere (microParticles, Berlin, Germany) was attached to a soft tipless cantilever SD-qp-CONT-TL (NanoWorld, Neuchâtel, Switzerland) using epoxy resin. A petri dish containing either distilled water for calibration or cell culture was placed on a motorized stage preheated to 37 °C. Before each experiment, the laser reflection sum was maximized, and the laser detector was centered. Immediately after, probe sensitivity and stiffness were determined using the thermal noise method in Bruker-JPK software. Typical AFM settings were setpoint in the range of 0.2–0.8 nN relative to the baseline to maintain indentation depths up to 2.8 µm, *Z*-length at 10 µm, recording speed of 20 µm s^{-1}, and sample rate at 5 kHz. Each force map consisted of 64×64 or 32×32 force–distance curves, covering an area of single or multiple cells, and Young’s modulus was calculated from the force–distance curves by fitting the DMT modelS[S3] in AtomicJ software.[S4]

Scanning Electron Microscopy. PMMA 100 nm particles and PS200-900 particles were recorded on a Teneo VolumeScope microscope using an operation voltage of 10 kV.

Isolation of RNA and PCR analysis. Total RNA was isolated using a High Pure RNA Isolation Kit, according to the manufacturer’s protocol. Complementary DNA was synthesized using the RT^2 First Strand Kit (SABiosciences Corporation, Frederick, USA). The expression levels of genes involved in ECM and cell adhesion were analyzed using RT^2 Profiler PCR Arrays (Qiagen). RT-PCR was performed on the LigthCycler 480 Real-Time PCR System (Roche, Basel, Switzerland), and the cycling parameters were 1 cycle at 95 °C for 10 min, followed by 45 cycles at 95 °C for 15 s and 60 °C for 1 min. Normalization of gene expression levels was performed using an internal panel of housekeeping genes provided by the manufacturer. Genes with a high coefficient of variation among replicas or very low expression (35 < Ct < 40) were excluded from the analysis. The results are presented as heatmaps of quantification cycles (Ct) and graphs of mean ± standard deviation (s.d.) values of fold regulation, based on three samples per experimental condition.

Cell transfection. Cells were transfected using lipofectamine 3000. The plasmids YAP1 (S6A)-V5 in pLX304 and pLX304 were obtained from Addgene as gifts from William Hahn (plasmid 42562) and David Root (plasmid 25890), respectively.[S5] shYAP (sc-38637-SH) plasmid was purchased from Santa Cruz Biotechnology. HEK 293T cells were seeded onto a 6-well plate and transfected 24 h later with a preincubated mixture containing 250 µL

Opti-MEM, 2.5 ng DNA or 2 ug of shRNA plasmid, 7.5 µL lipofectamine 3000, and 5 µL P3000 reagent, added dropwise into each well. After 12 h, fresh medium was added, and cells were allowed to grow for another 8 h. The cells were then detached, seeded in a 24-well plate at a density of 200,000 cells/well, and cultured for an additional 12 h prior to incubation with nanoparticles.

Generation of YAP reporter cell line. To produce second-generation lentiviral particles, HEK 293T cells were cotransfected with a three-plasmid combination: pMD2.G (Addgene #12259), psPAX2 (Addgene #12260), and pLL3.7 FLAG-YAP1-TEAD-P-H2B-mCherry (Addgene #128327) using FuGENE. The cell culture media was collected for 3 days, centrifuged and filtered to exclude cell debris, and pooled. Cells were incubated with media enriched in viral particles and supplemented with 10 µg mL^{-1} polybrene (Santa Cruz Biotechnology) for 6 h. The culture maintenance media was replaced daily and after 1 week, and cells were sorted for mCherry-positive cells on a MoFlo Astrios EQ (Beckman Coulter).

Nanoparticle internalization studies. The day before the experiments, 50,000 or 200,000 cells were seeded in 500 µL of medium onto a 24-well plate. After 18 h, the cells were incubated with nanoparticles diluted at the desired concentration in the supplemented medium for 4 h (or according to the time indicated for each experiment), with 500 µL of nanoparticle suspension added per well. The samples were then processed for downstream flow cytometry and confocal analysis according to the following protocols: for cytofluorimetric analysis, WT or YAP −/− HEK 293T cells were cultured on 24-well plates and left for 12 h to adhere, then incubated with PMMA100, Doxo-NP, PS200 or PS900 for 4 h. For the experiments with gold nanoparticles (AuNPs), the cells were incubated for 12 h with the particles at ratio of 5,000 and 25,000 NPs/cell, for the 50 nm AuNPs, and ratio of 2,500 and 5,000 NPs/cell, for the 100 nm AuNPs. For the exocytosis experiments after 4 h incubation with the particles, the cells were washed and seeded in fresh media for up to 48 h post-treatment. For the experiments with the XMU-MP1, cells were treated with the desired concentration of inhibitor for 4 h, before the addition of PS200 or PS900 to the medium containing XMU-MP1 for additional 4 h. The medium was removed, 200 µL of TrypLE Express enzyme was added to each well for 5 min, and the cells were detached and washed three times until no particle signal was detected in the supernatant. The samples were analyzed using the FACSAria II flow cytometer (Becton Dickinson, USA), and plots were prepared with FlowJo software V10 (Tree Star, USA). For confocal laser scanning microscopy analysis, following incubation of cells with nanoparticles, the cells were detached, washed three times with phosphate-buffered saline (PBS), and left to adhere to a µ-slide 8-well glass bottom dish. After 4 h, the cells were processed according to the immunohistochemistry protocol described below.

Immunohistochemistry and image analysis. For immunohistochemistry, 40,000 cells per well were seeded onto a µ-slide 8-well glass bottom dish for 24 h. After each relevant experiment, the medium was removed and the cells were washed with PBS. Before staining, the cells were fixed with 200 µL 4% paraformaldehyde in PBS for 15 min at room temperature, permeabilized with 0.1% Triton X-100 for 5 min, and then blocked with 2.5% bovine serum albumin (BSA) in PBS for 30 min. The primary antibodies were added in 200 µL PBS-BSA 2.5% solution and incubated for 2 h at room temperature or overnight at 4 °C. The secondary Alexa fluorochrome-conjugated antibodies were then added, and the cells were incubated in PBS. F-actin was stained with Pha-488 or Pha-647, the membrane was stained with WGA-488 or WGA-647, and the nuclei were counterstained with DAPI or Hoechst. Spheroids were stained according to the same procedure. Some of the samples were embedded in Mowiol

reagent. The samples were visualized with a Zeiss LSM 780 or Leica TCS SP8 X confocal microscope. *Z*-stacks were acquired with the optimal interval suggested by the software, and a maximum intensity algorithm was applied. Images were analyzed using ImageJ (http://rsb.info.nih.gov/ij/). The primary antibodies used were mouse anti-YAP (4912, Cell Signaling Technology) and mouse anti-Vinculin (V9131, Merck), diluted at a ratio of 1:200.

Western blotting. Cells were lysed with RIPA buffer containing 1% protease and phosphatase inhibitor cocktails on ice, and then centrifuged at 14,000 *g* for 15 min at 4 °C. The supernatants were stored at 80 °C. Protein concentrations were determined using the bicinchoninic acid protein assay, and 20 µg of protein per sample was loaded onto 10% polyacrylamide gels and run at 100 V. Proteins were transferred to a polyvinylidene difluoride membrane using the Trans-Blot Turbo transfer system (Bio-Rad). The membranes were blocked with 5% BSA in tris-buffered saline - 0.1% Tween (TBST) and incubated with primary antibodies diluted in 5% BSA in TBST overnight at 4 °C. The membranes were then probed with HRP-linked secondary antibodies for 1 h at room temperature. Chemiluminescence was detected using the ChemiDoc imaging system (Bio-Rad), and band intensities were quantified using the Bio-Rad Image Lab software. The primary antibodies used were rabbit anti-YAP (1:1000), mouse anti-β-tubulin (1:1000), rabbit anti-MST1 (1:500), rabbit anti-MST2 (1:500), rabbit anti-pYAP S397 (1:500), rabbit anti-pMOB1 T35 (1:500), mouse anti-GAPDH-peroxidase (1:25,000); anti-rabbit IgG, HRP-linked antibody (1:1000).

RNA-seq. For RNA-seq and data analysis, libraries were prepared using the NEBNext Ultra II Directional RNA Library Prep Kit for Illumina, with the NEBNext Poly(A) mRNA Magnetic Isolation Module and NEBNext Multiplex Oligos for Illumina (Dual Index Primers Set 1). The kits were utilized according to the manufacturer's instructions, with 200–300 ng of total RNA serving as the input for library preparation. Sequencing was conducted on an Illumina NextSeq 500 using the NextSeq 500/550 High Output v2 kit (75 cycles). Single-end 75bp sequencing was performed across multiple runs to ensure that all samples had at least 30 million passing filter reads. Fastq files were generated using bcl2fastq software without any trimming. The quality of the raw sequencing data was evaluated using FastQC (https://www.bioinformatics.babraham.ac.uk/projects/fastqc/) and aligned to the hg38 reference genome using the TopHat2 aligner. Raw gene counts were calculated from reads mapped to exons and summarized by genes using the Ensembl 90 reference gene annotation (Homo sapiens GRCh38.p10, GTF) with HTSeq. Differential gene expression was identified using the DESeq2 Bioconductor package, with genes considered differentially expressed if the Benjamini–Hochberg adjusted P-value was ≤ 0.05 and log2FC was ≥ 1. Biological term classification and gene cluster enrichment analysis were conducted using the clusterProfiler package, and all computations were carried out using BioJupies.[S6] Enrichment analysis and ranking were performed using Enrichr, and the most significant annotations were downloaded from the available repository (https://maayanlab.cloud/Enrichr/).[S7-9] The GO categories were downloaded from the AmiGO 2 repository (https://amigo.geneontology.org).

Image processing. Images were processed using FIJI, Imaris (version 10.0.1), and LAS X softwares. Using Imaris, stack images were first converted to imaris files with ImarisFileConverter, and 3D reconstruction was performed with the "volume rendering" function. Optical slices were obtained with the "orthoslicer" tool.

Statistical analysis. Results are based on at least three replicates, with data presented as the mean ± s.d. Calculations were carried out using GraphPad Prism v. 6.0 (San Diego, USA). For single-cell analysis, a minimum

of 100 cells per sample were analyzed. The statistical tests used include the unpaired t-test with Welch's correction, multiple t-tests, Kruskal–Wallis one-way ANOVA followed by Dunn's multiple comparisons test, one-way ANOVA followed by Tukey's multiple comparisons test, and two-way ANOVA followed by Sidak's or Tukey's multiple comparisons test. The appropriate statistical test was applied as indicated in the figure captions for each experiment. A *P*-value of less 0.05 was considered statistically significant as denoted with asterisks (*$p < 0.05$, **$p < 0.01$, ***$p < 0.001$).

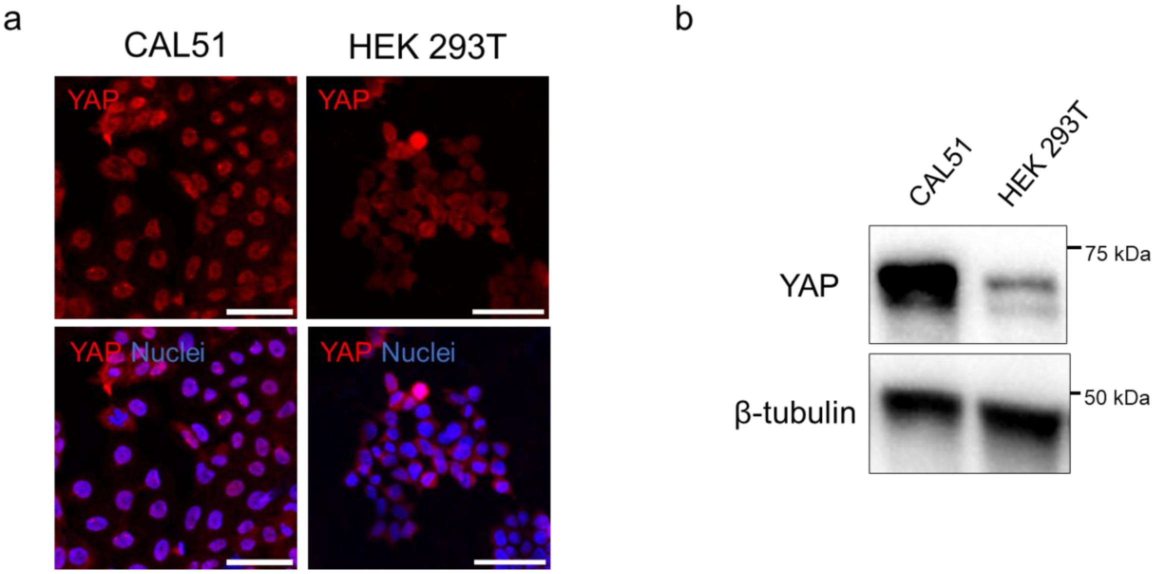

Figure S1. (a) Confocal images of CAL51 and HEK 293T cells stained for YAP (Alex Fluor 555, red) and DAPI (blue). Scale bars: 50 μm. (b) Western blot showing the levels of YAP in CAL51 and HEK 293T cells. YAP band in HEK 293T cells as shown in Figure 1a. β-Tubulin was used for protein loading normalization. For a direct comparison with our previous work, here we show the data obtained from CAL51 cells, as previously shown,[S1] with those obtained from HEK 293T cells.

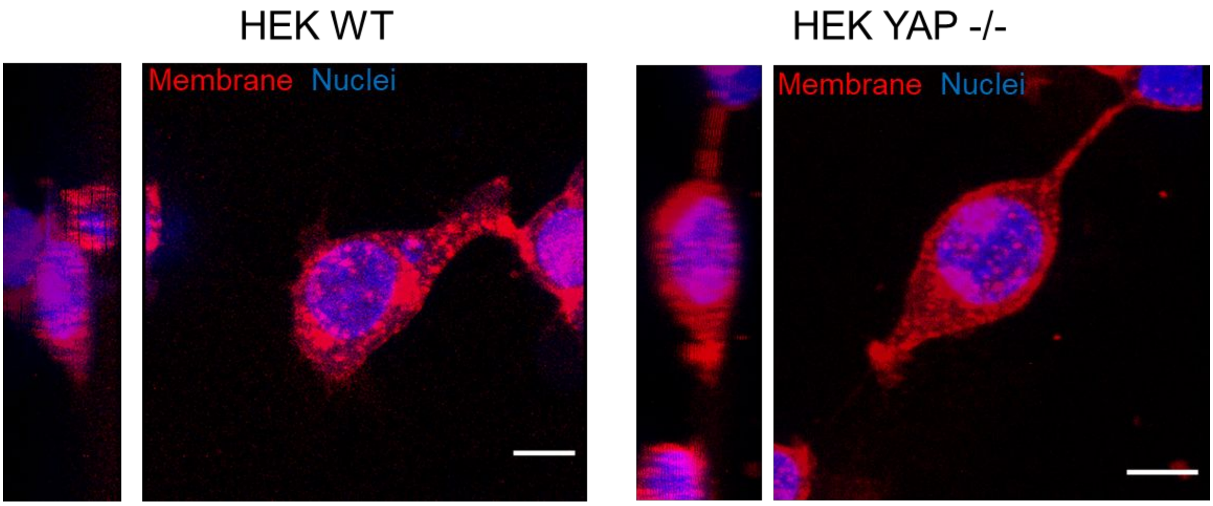

Figure S2. 3D reconstruction of WT (left) and YAP −/− (right) HEK 293T cells. The lateral views are presented. The cells are stained with DAPI (blue) and WGA-647 (red). Scale bar is 10 μm.

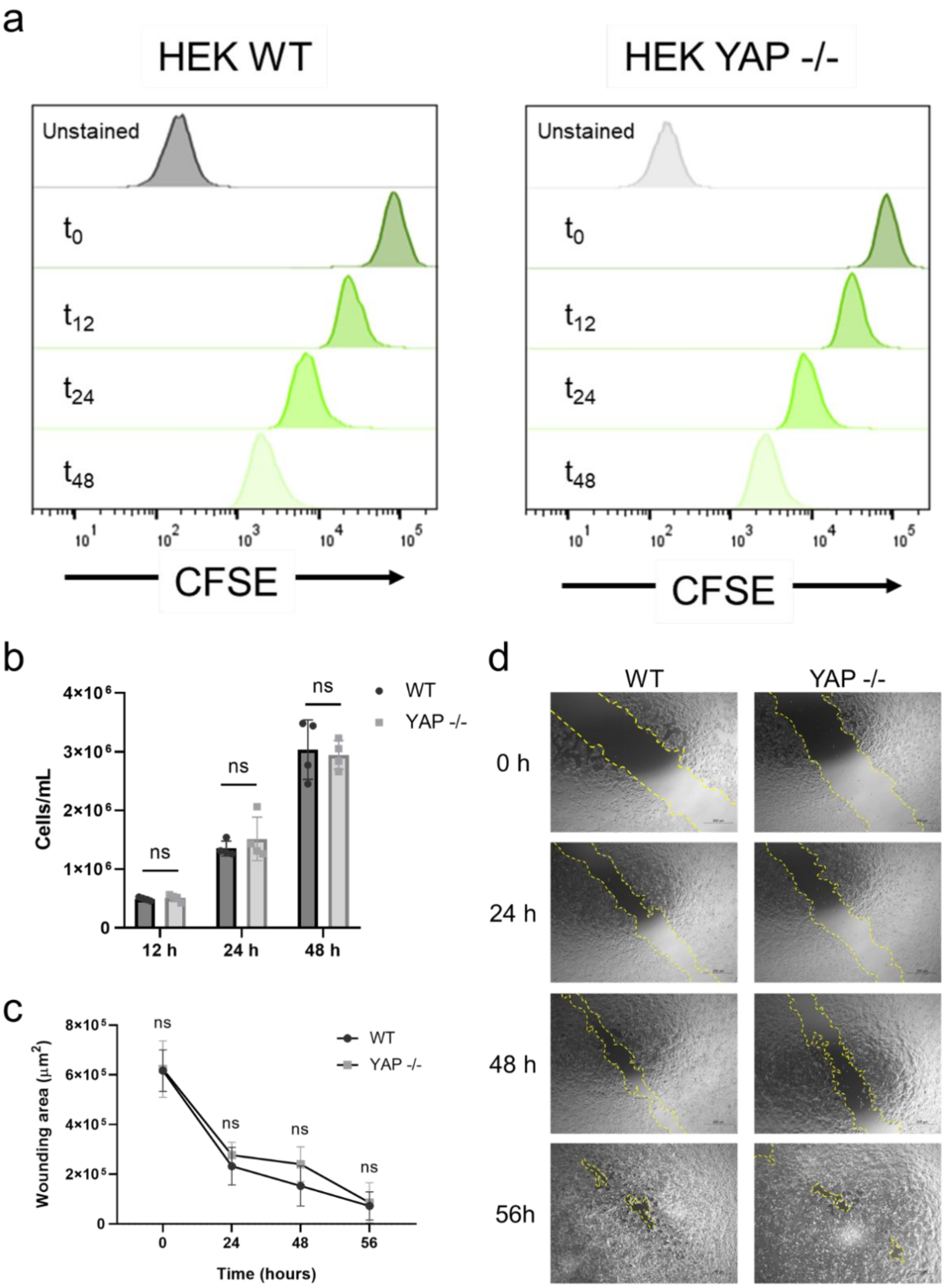

Figure S3. a) Histogram of the CFSE assay for WT (left) and YAP -/- (right) HEK 293 T cells 12, 24, and 48 hours after seeding. Unstained cells are presented in dark grey (WT) and light grey (YAP -/-), cells right after seeding are presented in dark green, and cells after 12, 24 and 48 hours of seeding are presented in shades of green. b) Cell number measured via the trypan blue assay 12 hours after WT (dark grey) and YAP -/- (light grey) cell seeding. Statistical analysis was performed by two-way ANOVA followed by Tukey's multiple comparisons test; n = 4; ns indicates non-significant. c) Wound closure expressed as the remaining area (expressed in μm^2) uncovered by WT (dark grey) and YAP -/- (light grey) cells. Statistical analysis was performed by two-way ANOVA followed by Sidak's multiple

comparisons test; n = 4; ns indicates non-significant. d) Representative phase-contrast microscope images showing the area covered by the WT (left) and YAP -/- (right) cells at 0, 24, 48 and 56 h after wounding. Magnification is 4×. Yellow dashed line indicates the remaining wound area at the different time points. Scale bar is 250 μm.

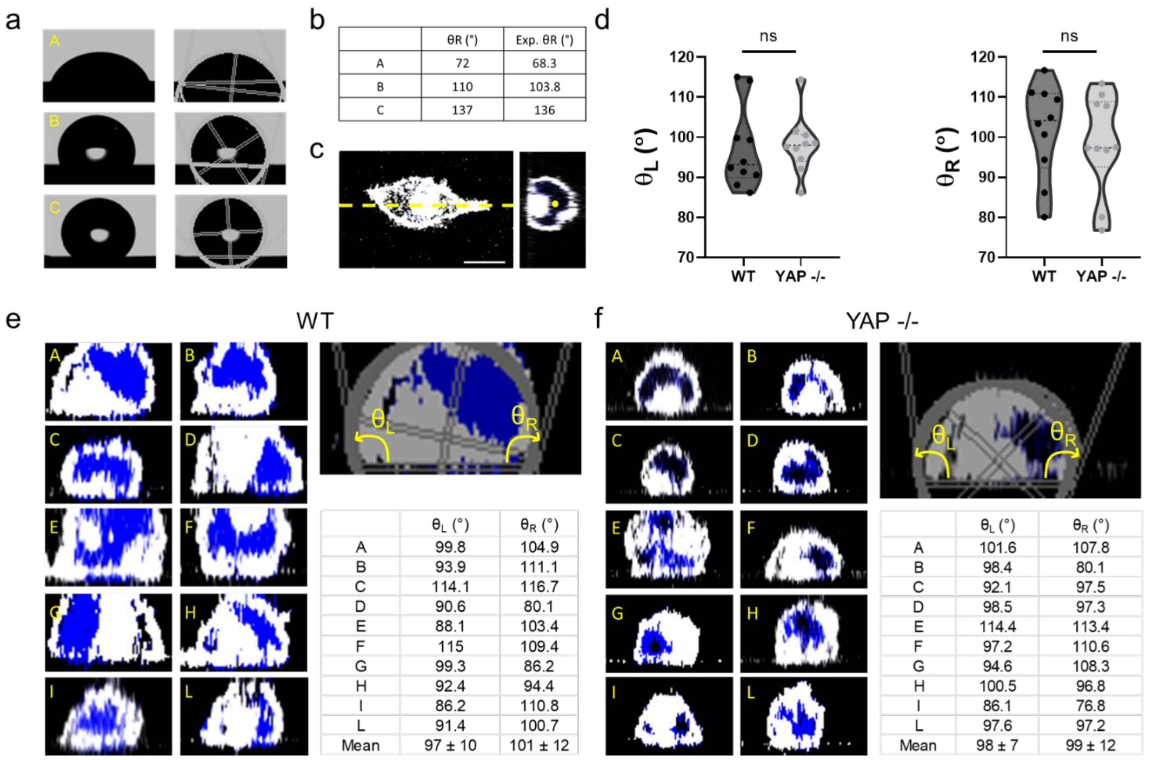

Figure S4. (a) Representative images of the contact angle for water drops used as reference for validating the "Contact angle" plugin implemented in Image J and used to measure the contact angle of HEK WT and YAP -/- cells. (b) θ right angle for the water drops of figure S4a as determined *via* "Contact angle" plugin or experimentally on a DataPhysics OCA 20 tensiometer. (c) The contact angle for HEK cells was determined considering their longitudinal section, as indicated by the dashed yellow line. (d) Violin plots of the θ left (θ_L, left) and θ right (θ_R, right) angles measured for HEK WT (dark grey) and YAP -/- (light grey) cells. Statistical analysis was performed by unpaired t-test. n = 10; ns indicates non-significant. (e) and (f) report the θ_L and θ_R calculated for each image (A-L) obtained from the 3D reconstructions of the confocal analysis of HEK WT (e) and YAP -/- (f) cells.

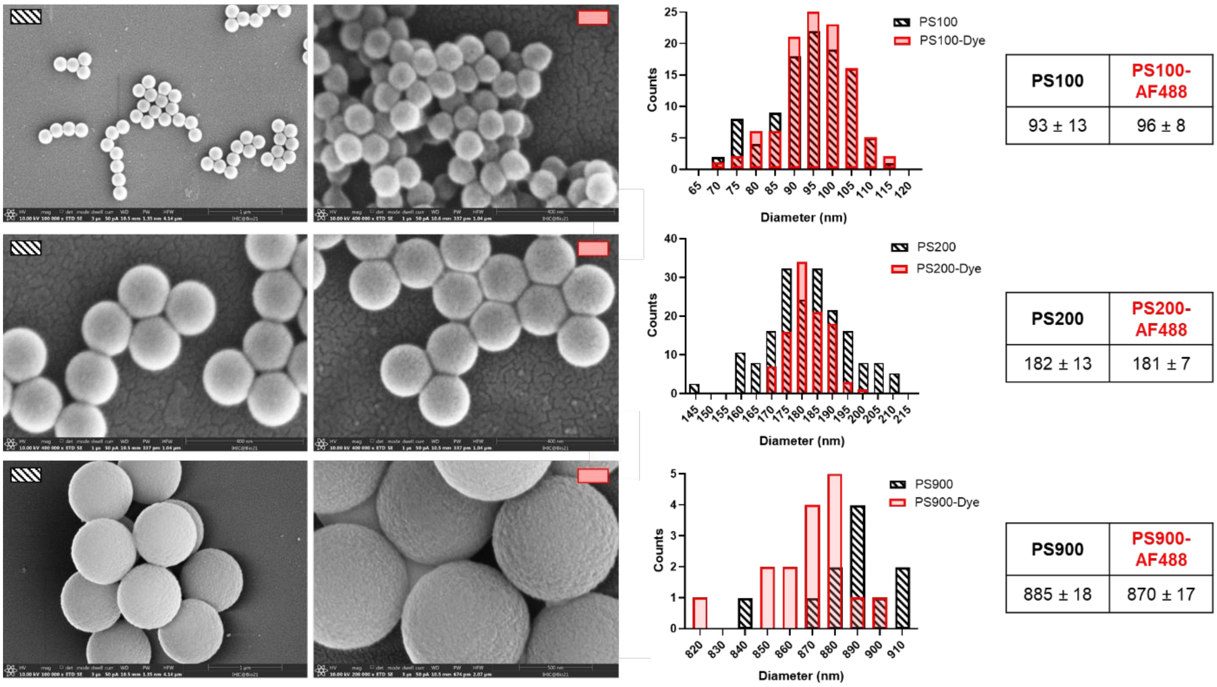

Figure S5. Representative TEM micrographs of 100 (top), 200 (middle) and 900 (bottom) nm polystyrene nanoparticles, non-functionalized (barred white box) and functionalized with Alexa Fluor 488-cadaverine dye (red box). The size distribution histogram and the relative size for each particle is shown. Scale bars: 1µm and 500 nm.

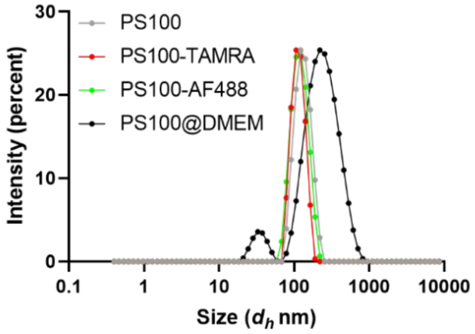

Sample	d_h (nm)	PDI	Z-potential (mV)
PS100	125 ± 2	0.05 ± 0.04	-22 ± 1
PS100-TAMRA	114 ± 2	0.04 ± 0.02	-29 ± 2
PS100-AF488	114 ± 0.5	0.04 ± 0.03	-46 ± 3
PS100@DMEM	163 ± 2	/	/

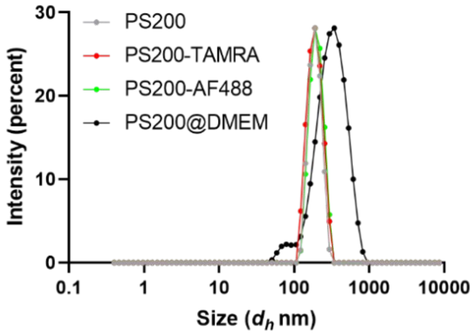

Sample	d_h (nm)	PDI	Z-potential (mV)
PS200	188 ± 2	0.06 ± 0.03	-35 ± 2
PS200-TAMRA	185 ± 2	0.01 ± 0.001	-38 ± 2
PS200-AF488	194 ± 3	0.04 ± 0.04	-28 ± 1
PS200@DMEM	278 ± 2	/	/

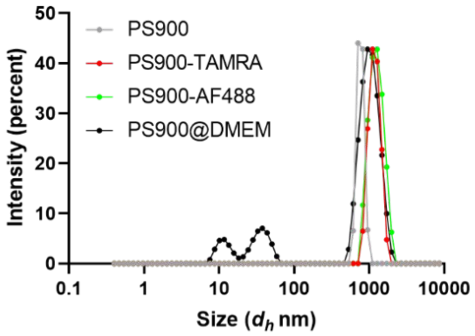

Sample	d_h (nm)	PDI	Z-potential (mV)
PS900	1238 ± 127	0.3 ± 0.06	-23 ± 2
PS900-TAMRA	1115 ± 40	0.06 ± 0.05	-36 ± 2
PS900-AF488	1216 ± 17	0.195 ± 0.04	-25 ± 1
PS900@DMEM	1146 ± 160	/	/

Figure S6. DLS graphs showing size weighted by intensity of PS100 (top), PS200 (middle) and PS900 (bottom) in 10 mM PBS and DMEM supplemented with FBS 10% (black line and dots). The tables reporting hydrodynamic diameter (*dh*), polydispersity index (PDI) and Z-potential for each particle (non-functionalized, light grey line and dots; carboxytetramethylrhodamine-functionalized, red line and dots; Alexa Fluor 488 cadaverine-functionalized, green line and dots) are also shown.

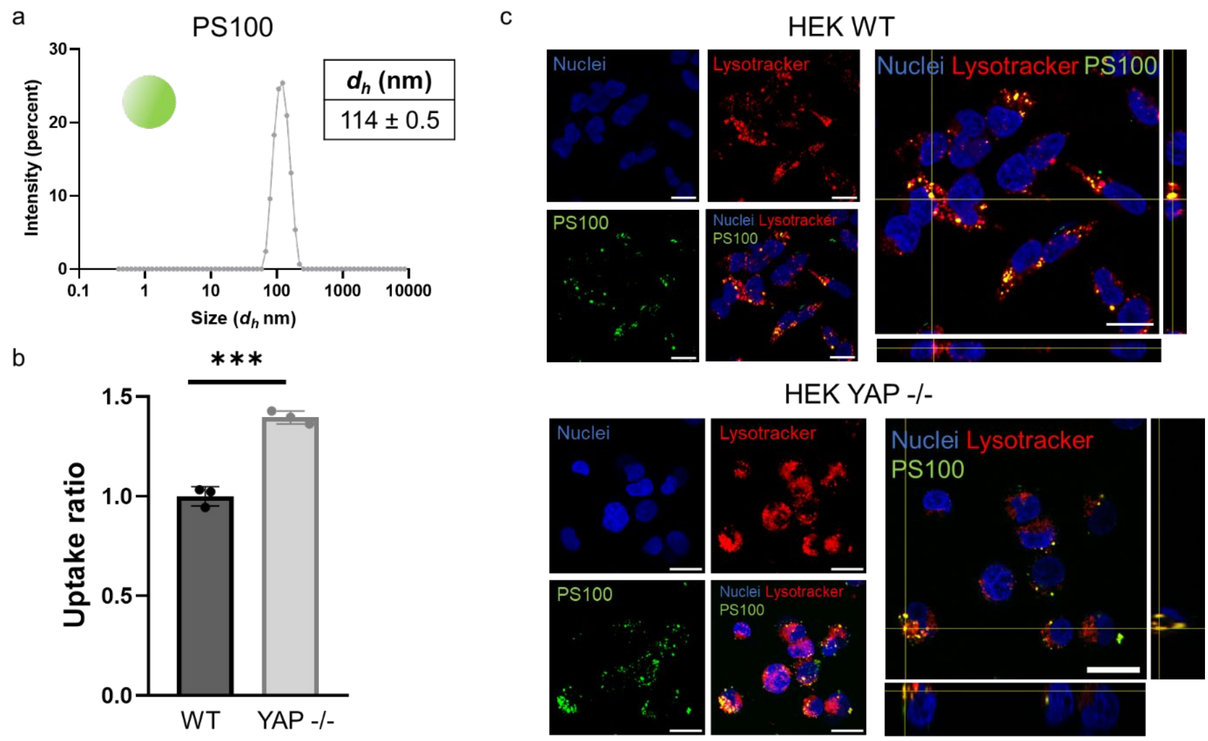

Figure S7. (a) DLS graph showing size weighted by intensity of PS100 in 10 mM PBS. (b) Uptake ratio of PS100 in HEK WT (dark grey) and YAP -/- (light grey) after 4-hours of incubation with the nanoparticles. Statistical analysis was performed by unpaired t-test. n = 3; ***p < 0.001. (c) representative confocal images of the intracellular localization of PS100 in HEK WT (top) and YAP -/- (bottom) cells after 4-hour incubation with the nanoparticles. Cells are stained with DAPI (blue) and Lysotracker (red). Orthogonal views of z-projection are also shown. Scale bar is 20 µm.

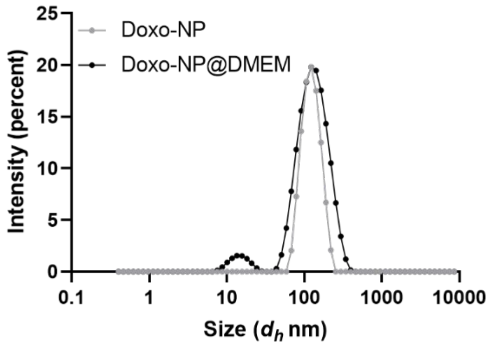

Sample	d_h (nm)	PDI	Z-potential (mV)
Doxo-NP	116 ± 1	0.1 ± 0.02	-2 ± 1
Doxo-NP@DMEM	104 ± 1	/	/

Figure S8. (a) DLS graph showing size weighted by intensity of Doxo-NP in 10 mM PBS (light grey line and dots) or DMEM supplemented with 10% FBS (black line and dots), with the relative measured hydrodynamic diameter, polydispersity index (PDI) and Z-potential.

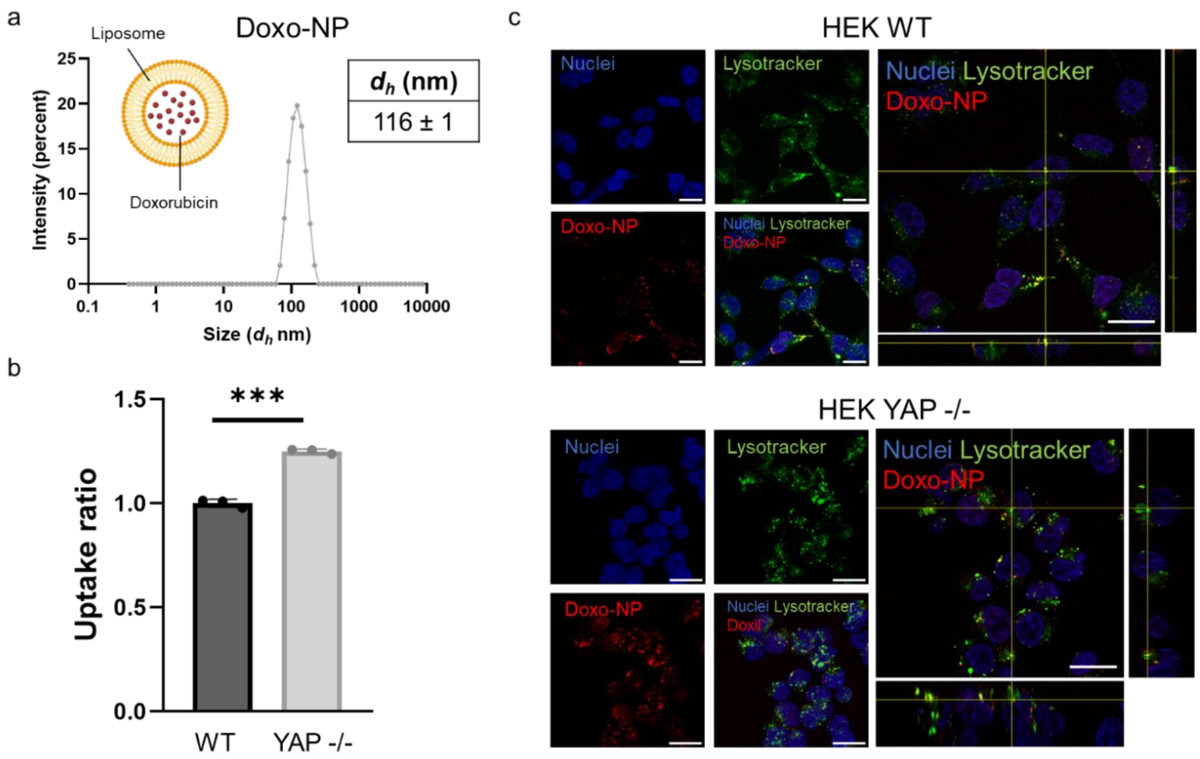

Figure S9. (a) DLS graph showing size weighted by intensity of Doxo-NP in 10 mM PBS. (b) Uptake ratio of Doxo-NP in HEK WT (dark grey) and YAP -/- (light grey) after 4-hours of incubation with the nanoparticles. Statistical analysis was performed by unpaired t-test. n = 3; ***$p < 0.001$. (c) representative confocal images of the intracellular localization of Doxo-NP in HEK WT (top) and YAP -/- (bottom) cells after 4-hour incubation with the nanoparticles. Cells are stained with DAPI (blue) and Lysotracker (green). Orthogonal views of *z*-projection are also shown. Scale bar is 20 μm.

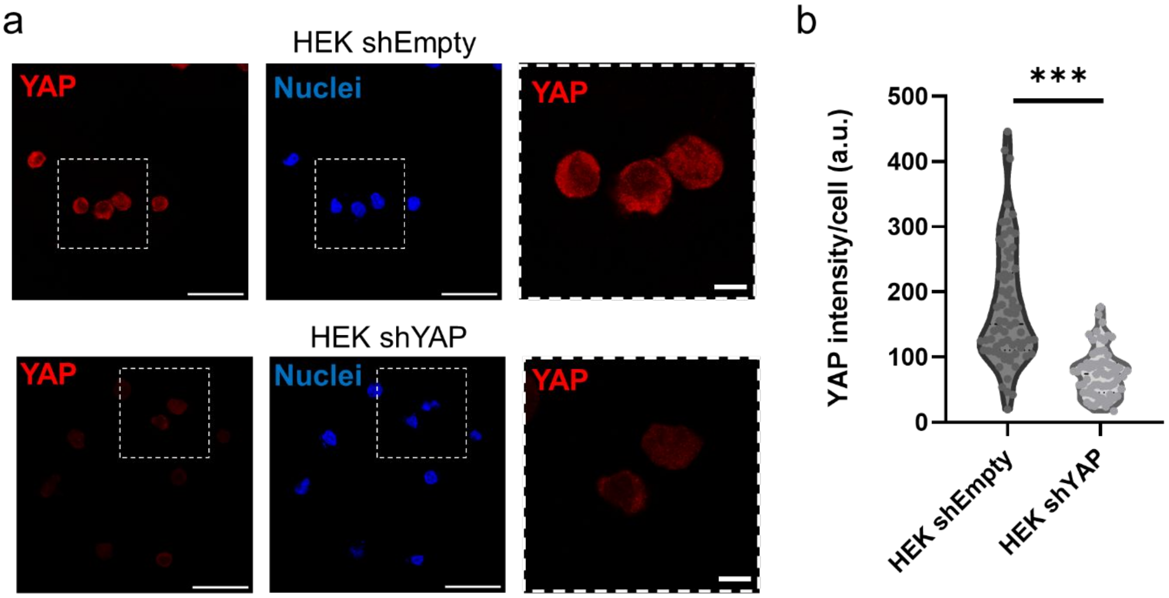

Figure S10. a) Confocal images of HEK WT cells transfected with an empty vector (HEK shEmpty) or a vector carrying a shRNA (HEK shYAP). Scale bars: 50 and 10 µm in the lower and higher magnification (regions highlighted with dashed white box) images, respectively. b) Violin plot of the signal intensity of YAP in the HEK shEmpty and HEK shYAP cells. Statistical analysis was performed using unpaired *t*-test with Welch's correction; $n > 60$; $***p < 0.001$.

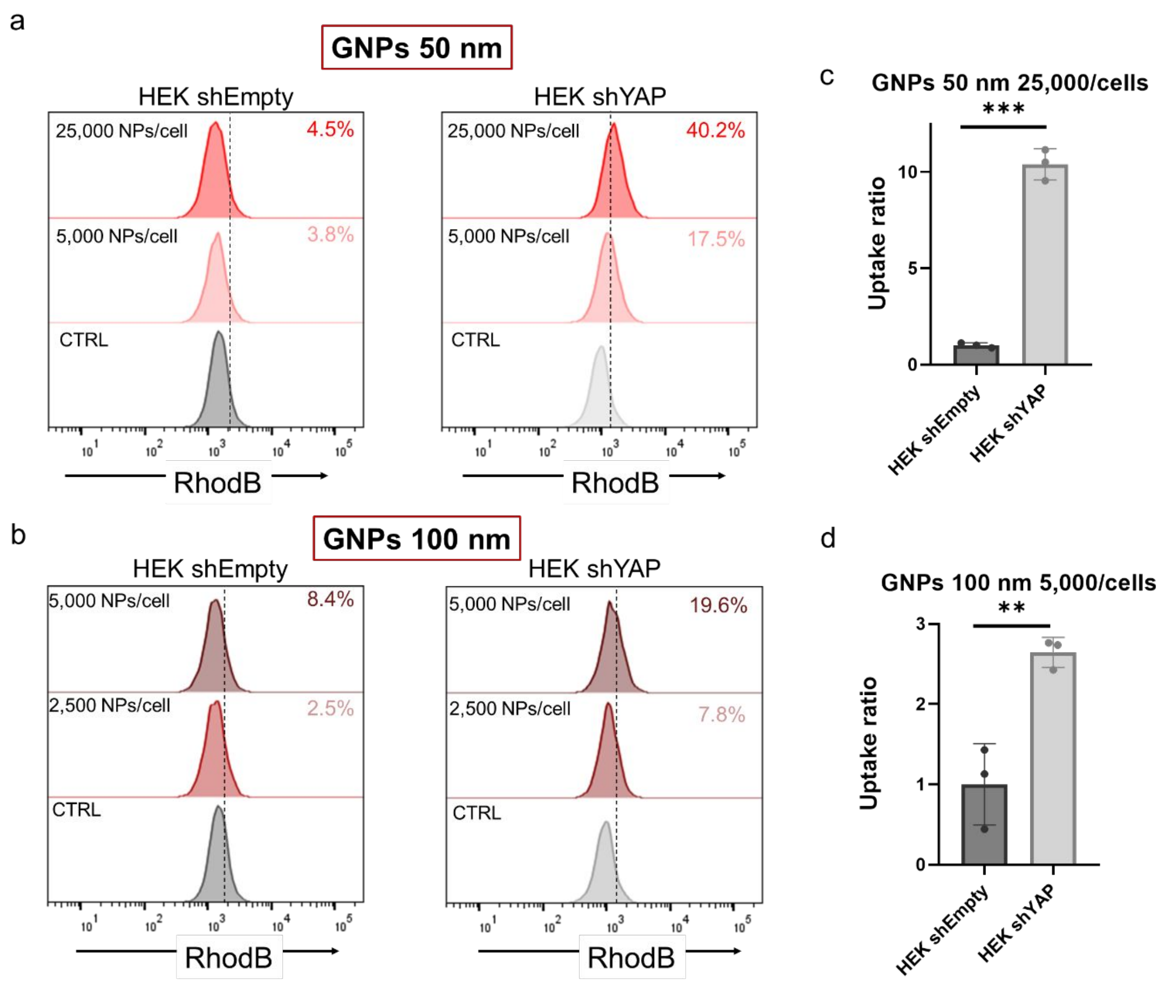

Figure S11. a) Representative histograms of shEmpty (left) and shYAP (right) HEK cells incubated for 12 hours with 50 nm Au@RhodB@MPN at the ratio of 25,000 NPs/cell (red) and 5,000 NPs/cell (light red), displaying the gating applied for rhodamine B-positive cells. b) Representative histograms of shEmpty (left) and shYAP (right) HEK cells incubated for 12 hours with 100 nm Au@RhodB@MPN at the ratio of 5,000 NPs/cell (dark red) and 2,5000 NPs/cell (red), displaying the gating applied for rhodamine B-positive cells. Uptake ratio of c) 50 nm Au@RhodB@MPN (c; 25,000 NPs per cell) and d) 100 nm Au@RhodB@MPN (d; 5,000 NPs per cell) in shEmpty (dark grey) and shYAP (light grey) HEK cells after 12-hours of incubation with the nanoparticles. Statistical analyses were performed by unpaired t-test. n = 3; ***$p < 0.001$.

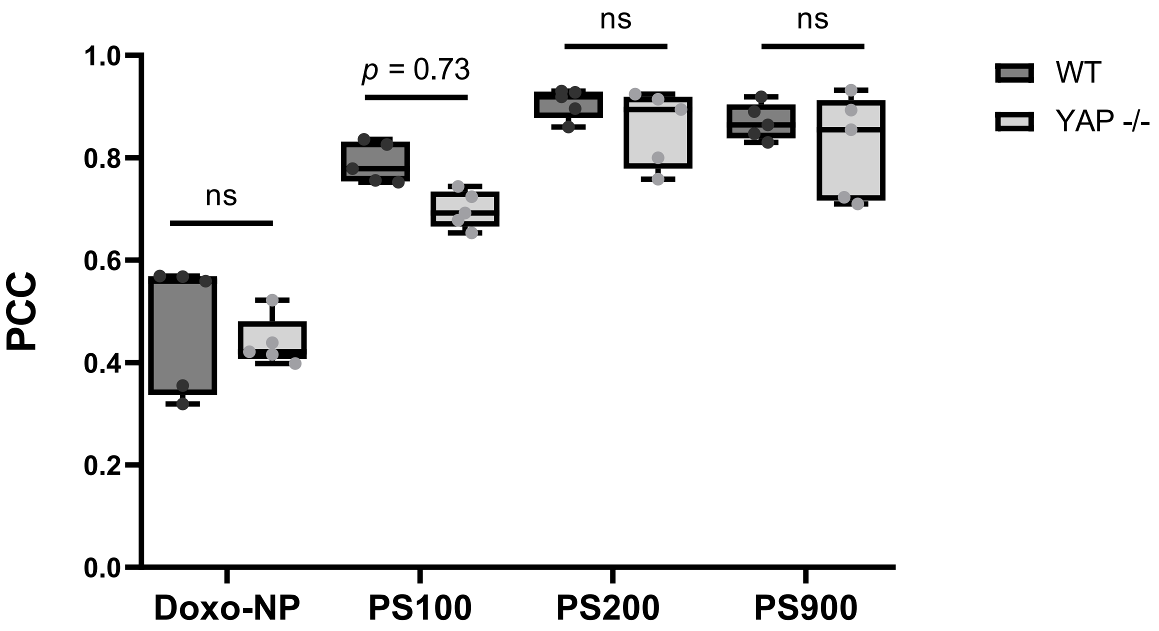

Figure S12. Colocalization of Doxo-NP and PS100 nanoparticles and PS200-900 particles with lysosomes in HEK WT (dark grey) and YAP -/- (light grey) assessed by Pearson's correlation coefficient (PCC) analyses. Statistical analyses were performed by two-way ANOVA followed by Sidak's multiple comparisons test. n = 5; ns indicates non-significant.

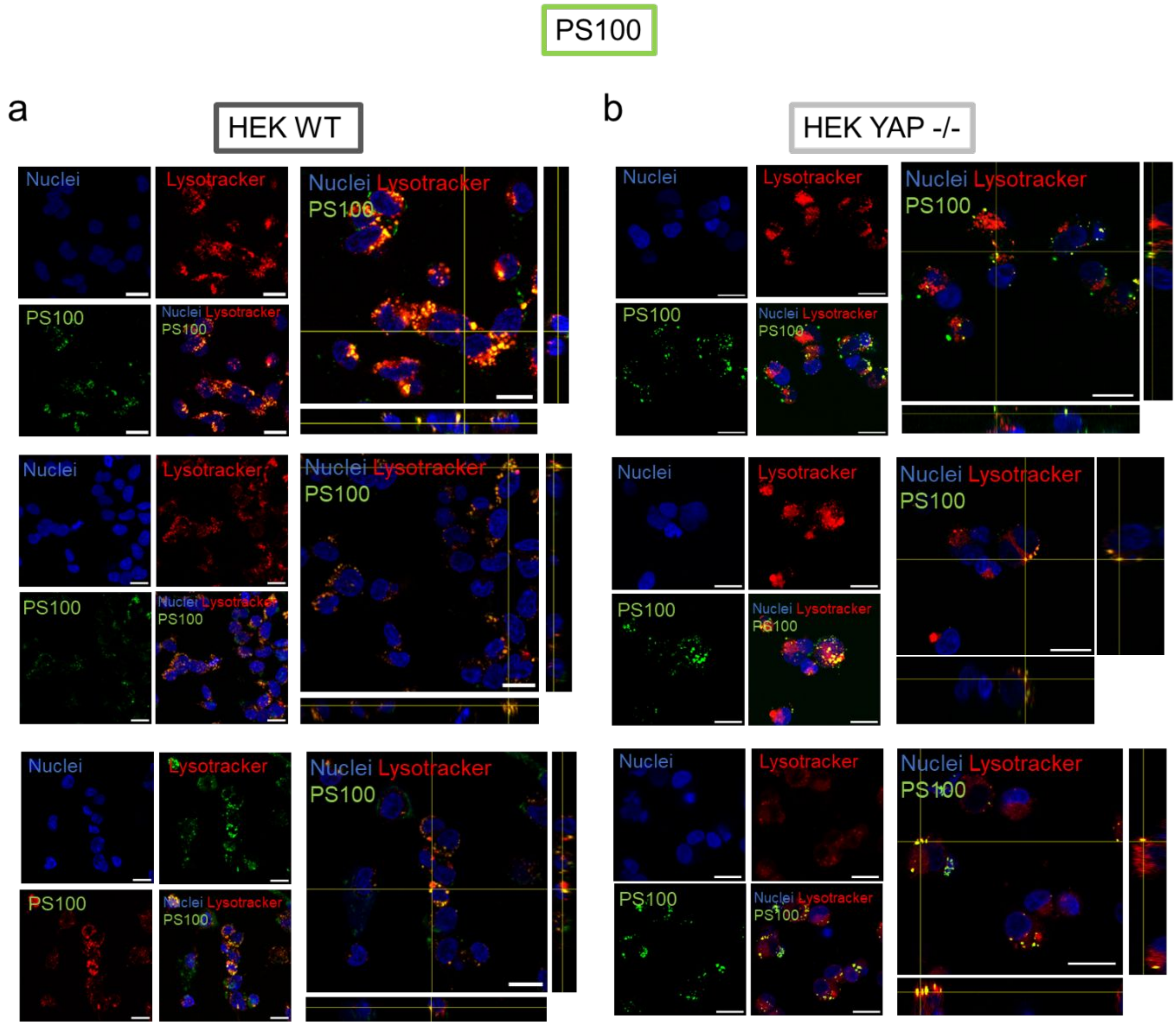

Figure S13. Representative confocal images of the intracellular localization of PS100 in HEK WT (a) and HEK YAP -/- (b) cells after 4-hour incubation with the nanoparticles. Cells are stained with DAPI (blue) and Lysotracker (green). Orthogonal views of *z*-projection are also shown. Scale bar is 20 µm.

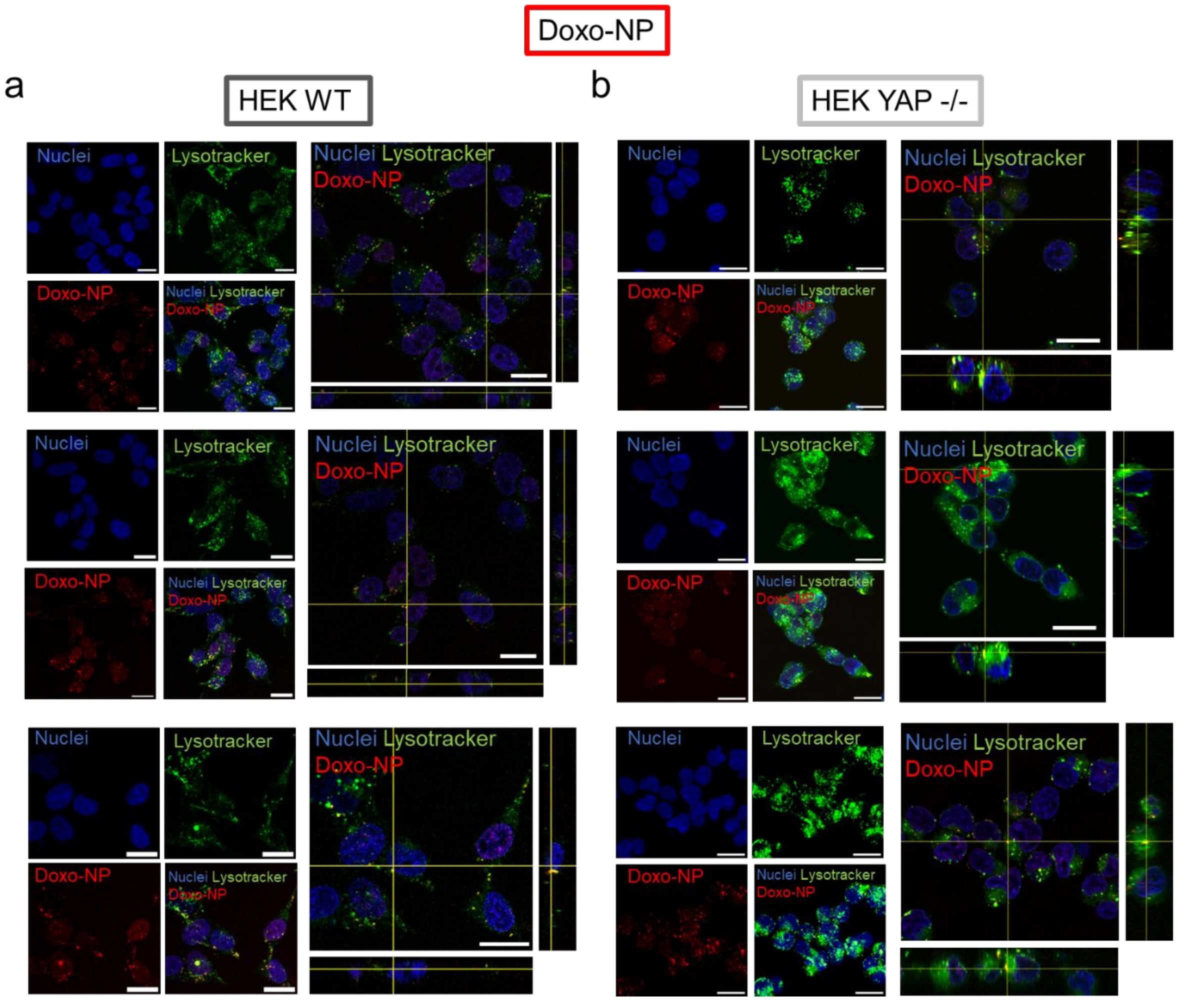

Figure S14. Representative confocal images of the intracellular localization of Doxo-NP in HEK WT (a) and HEK YAP -/- (b) cells after 4-hour incubation with the particles. Cells are stained with DAPI (blue) and Lysotracker (green). Orthogonal views of *z*-projection are also shown. Scale bar is 20 μm.

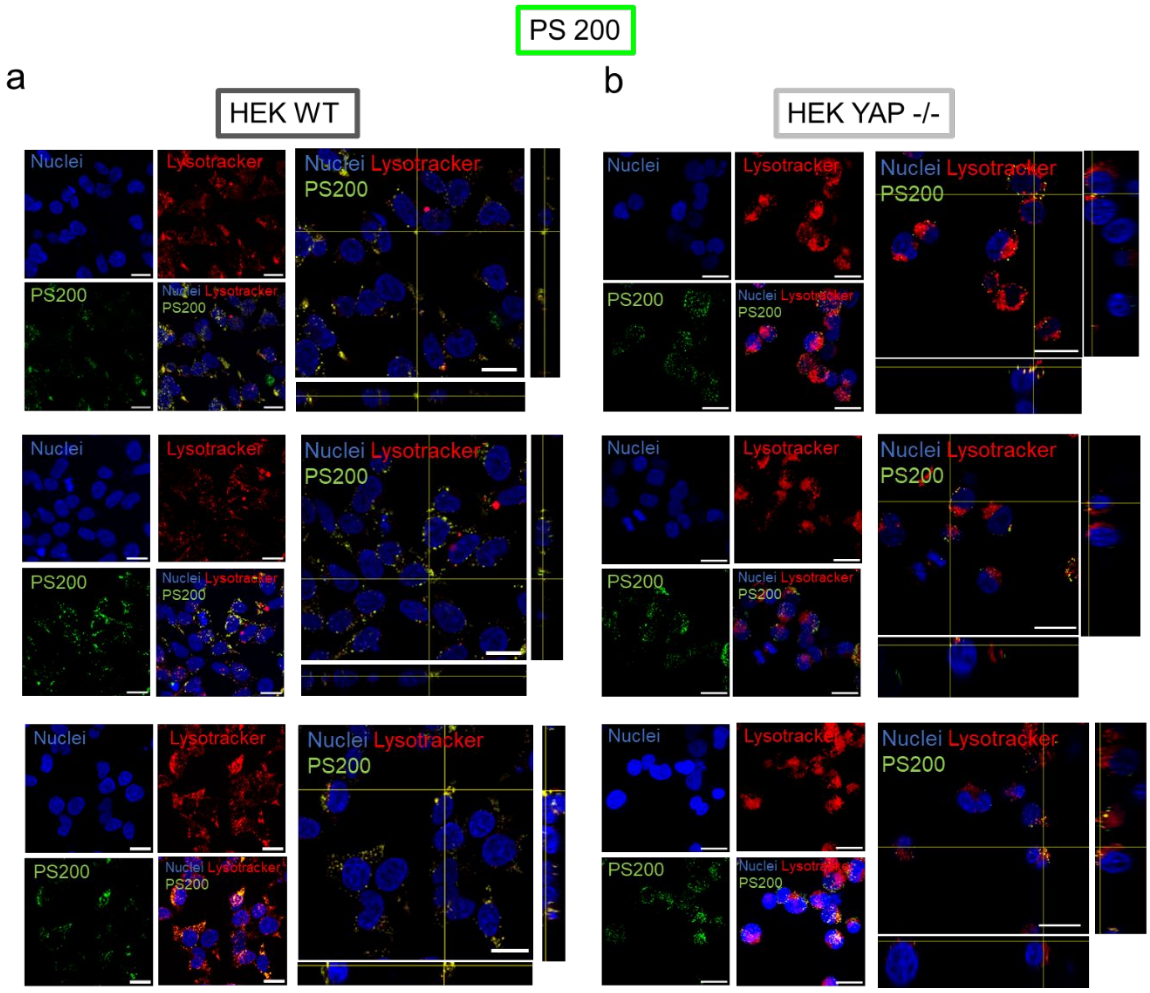

Figure S15. Representative confocal images of the intracellular localization of PS200 in HEK WT (a) and HEK YAP -/- (b) cells after 4-hour incubation with the nanoparticles. Cells are stained with DAPI (blue) and Lysotracker (red). Orthogonal views of *z*-projection are also shown. Scale bar is 20 μm.

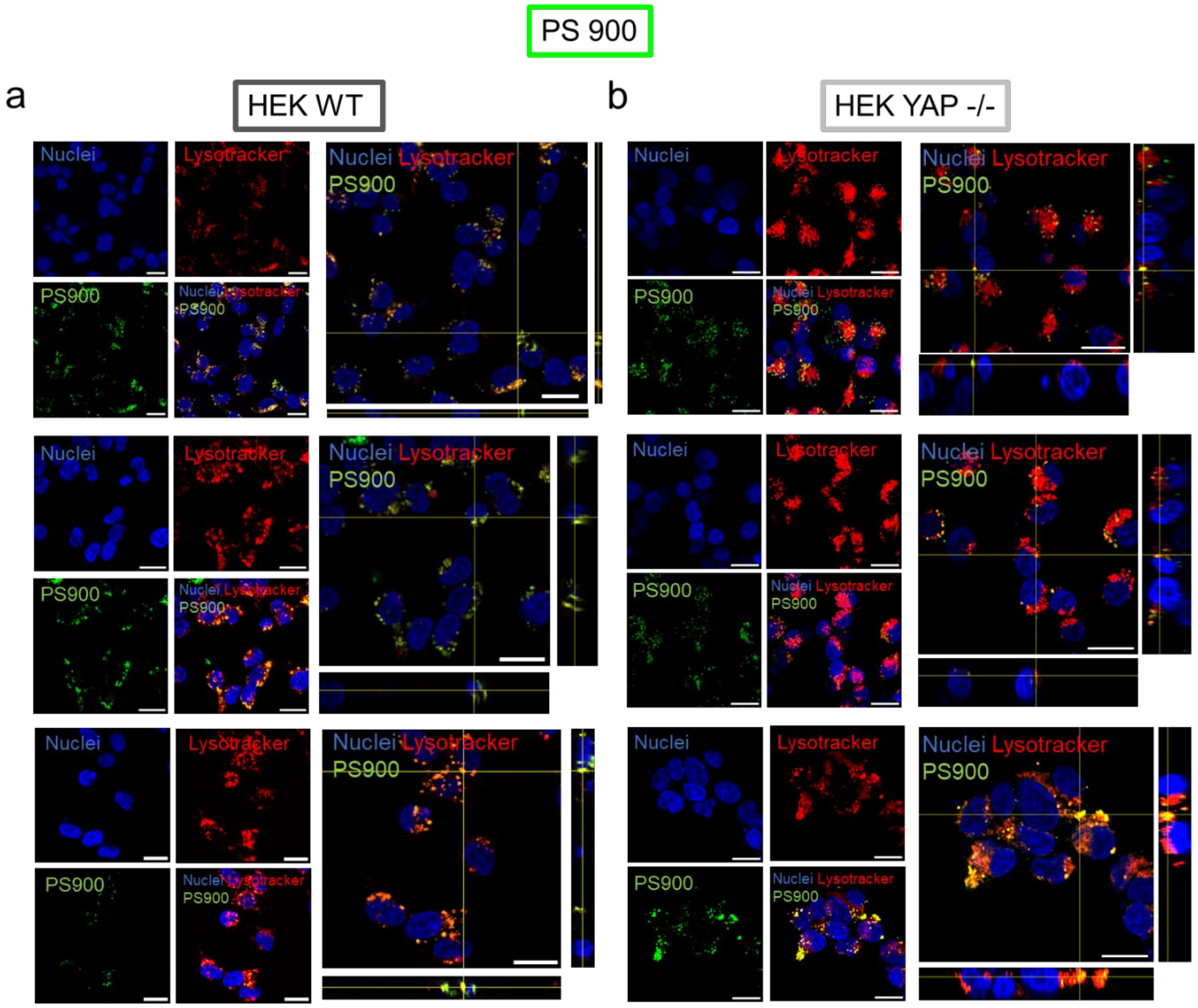

Figure S16. Representative confocal images of the intracellular localization of PS900 in HEK WT (a) and HEK YAP -/- (b) cells after 4-hour incubation with the particles. Cells are stained with DAPI (blue) and Lysotracker (red). Orthogonal views of *z*-projection are also shown. Scale bar is 20 µm.

PS200

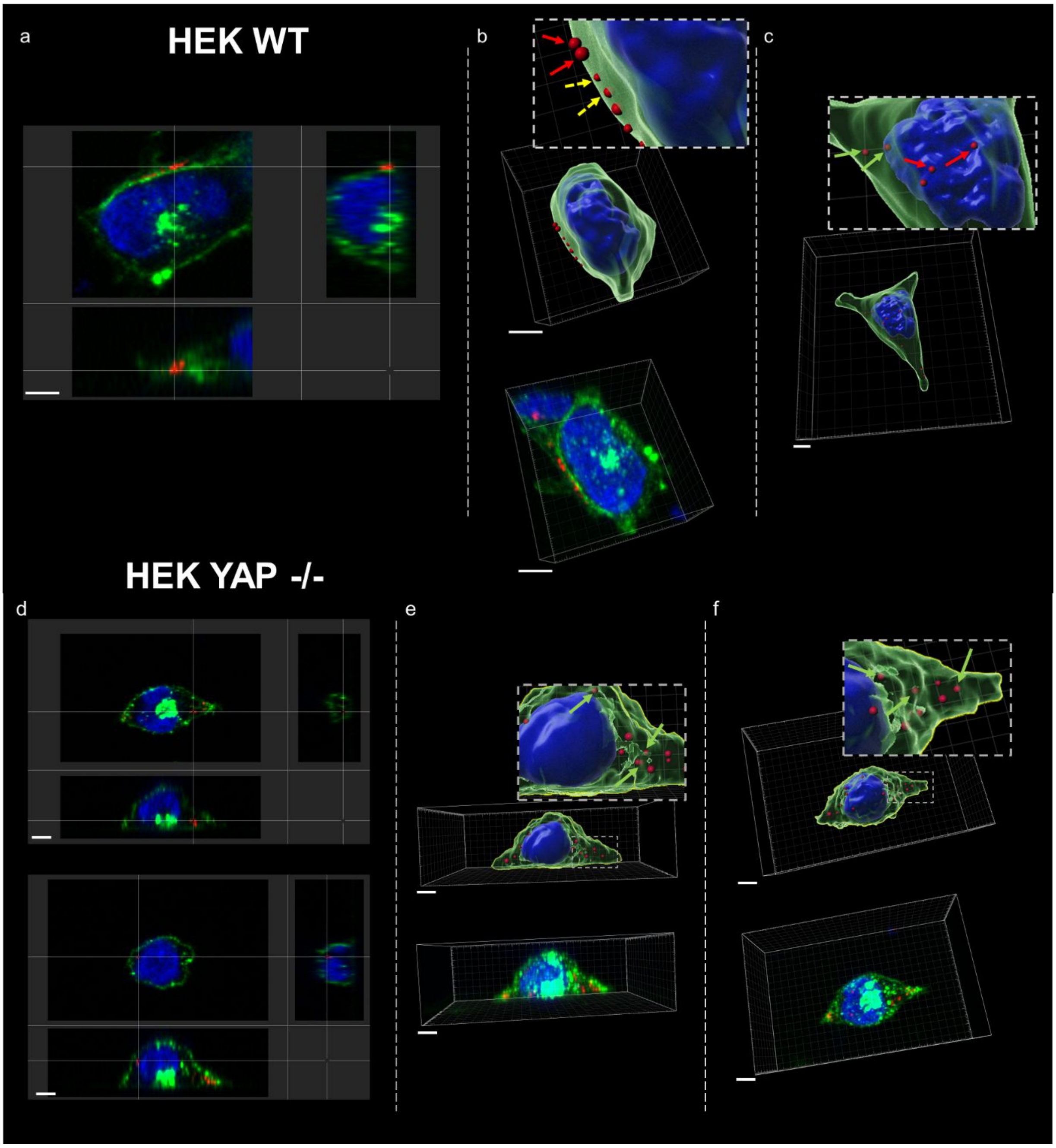

Figure S17. (a) Representative orthogonal views of *z*-projection of HEK WT cells after incubation for 4 h with PS200. (b) and (c) IMARIS 3D reconstructions, with the corresponding confocal 3D image (bottom). Cells are stained with DAPI (blue) and/or WGA 488 (green). Particles are shown in red. Red arrows indicate the particles bound to the membrane; yellow dashed arrows indicate particles colocalized with the membrane; green arrows indicate internalized particles. Scale bar is 5 µm. (d) Representative orthogonal views of *z*-projection of HEK YAP -/- cells after incubation for 4 h with PS200. (e) and (f) IMARIS 3D reconstructions, with the corresponding confocal 3D image (bottom). Cells are stained with DAPI (blue) and/or WGA 488 (green). Particles are shown in red. Red arrows

indicate the particles bound to the membrane; yellow dashed arrows indicate particles colocalized with the membrane; green arrows indicate internalized particles. Scale bar is 5 µm.

PS900

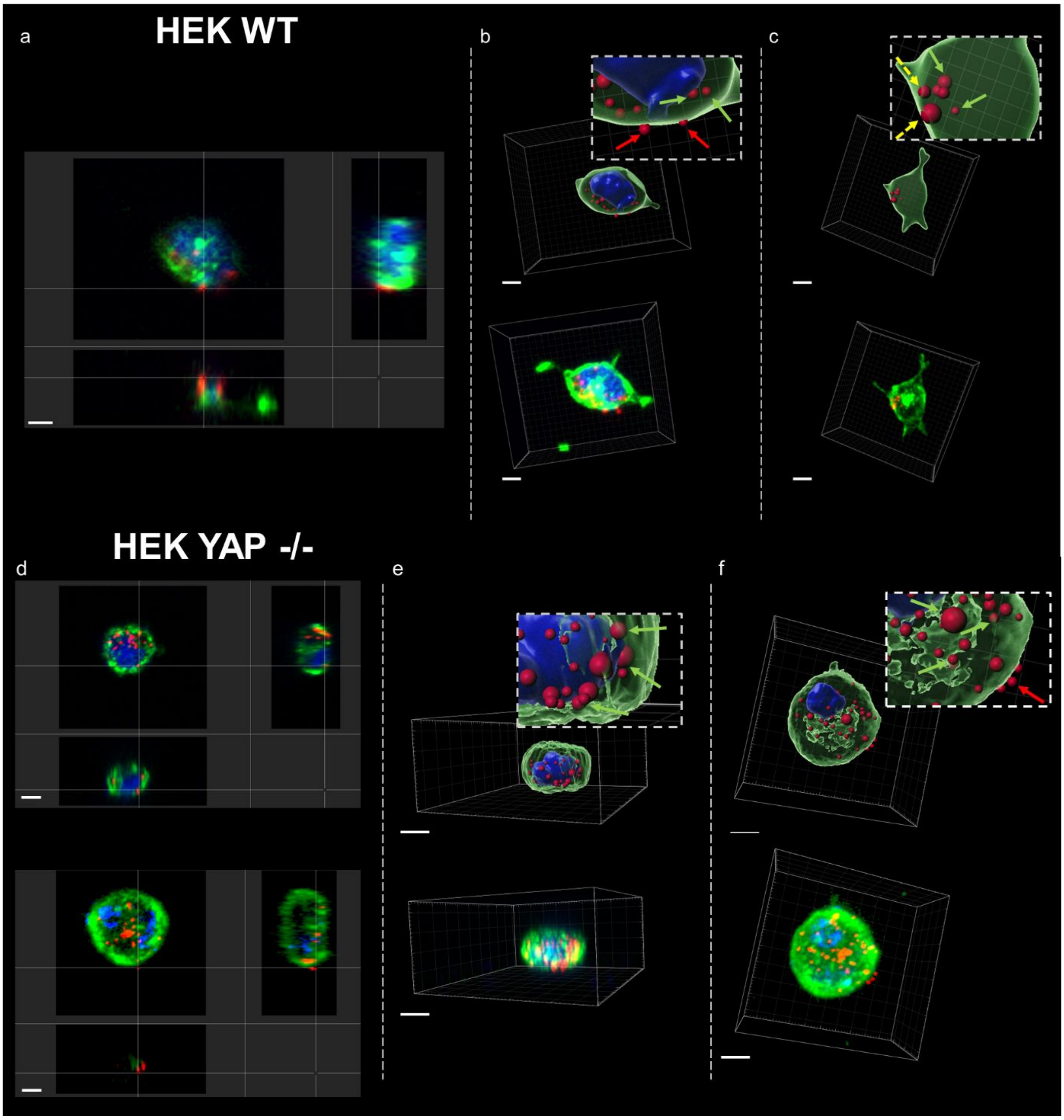

Figure S18. (a) Representative orthogonal views of *z*-projections of HEK WT cells after incubation for 4 h with PS900. (b) and (c) IMARIS 3D reconstructions, with the corresponding confocal 3D image (bottom). Cells are stained with DAPI (blue) and WGA 488 (green). Particles are shown in red. Red arrows indicate the particles bound to the membrane; yellow dashed arrows indicate particles colocalized with the membrane; green arrows indicate internalized particles. Scale bar is 5 µm. (d) Representative orthogonal views of *z*-projections of HEK YAP -/- cells after incubation for 4 h with PS900. (e) and (f) IMARIS 3D reconstructions, with the corresponding confocal 3D image (bottom). Cells are stained with DAPI (blue) and WGA 488 (green). Particles are shown in red. Red arrows

indicate the particles bound to the membrane; yellow dashed arrows indicate particles colocalized with the membrane; green arrows indicate internalized particles. Scale bar is 5 μm.

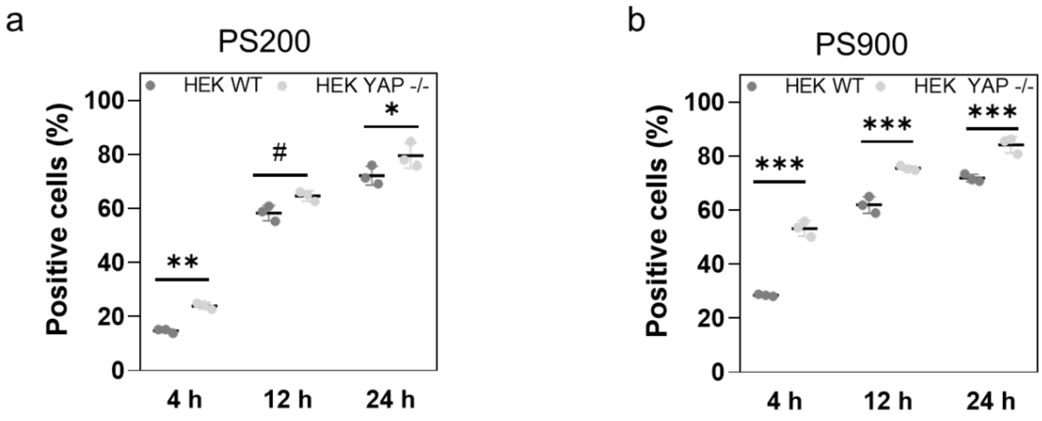

Figure S19. (a, b) Uptake of PS200 (a) and PS900 (b) in WT or YAP −/− HEK 293T cells following incubation for 4, 12, or 24 h. Statistical analysis was performed using two-way ANOVA followed by Tukey's multiple comparisons test; $n = 3$; ***$p < 0.001$; **$p < 0.01$; *$p < 0.05$; #$p = 0.0507$.

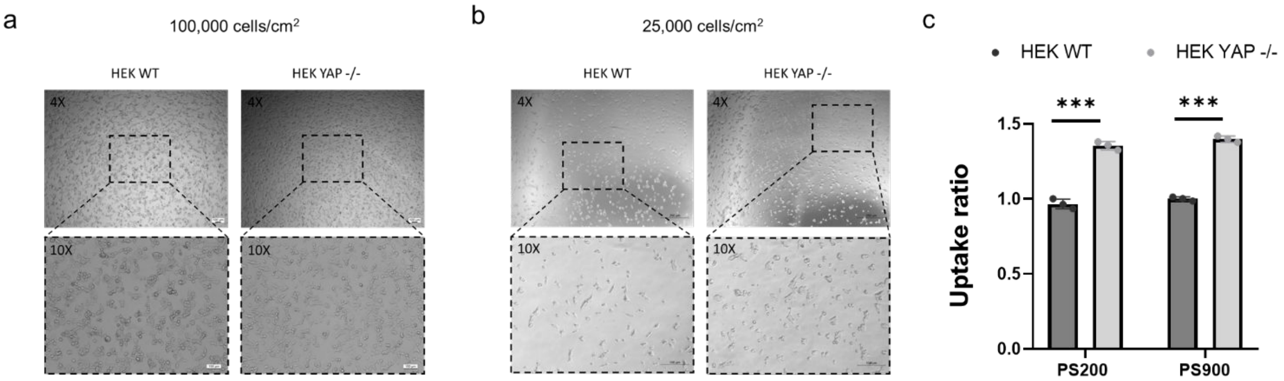

Figure S20. (a) and (b) representative brightfield images of WT and YAP -/- HEK cells seeded onto 24-well plates at densities of 100,000 cells/cm^2 (a) and 25,000 cells/cm^2 (b). Scale bar: 100 µm. (c) Uptake ratios of PS200 and PS900 in WT or YAP -/- HEK cells after incubation for 4 h at seeding density of 25,000 cells/cm^2. The data referring to a cell confluency of 100,000 cells/cm^2 can be found in Figure 2 of the main text. Statistical analysis was performed using two-way ANOVA followed by Sidak's multiple comparison test; n = 3; ***$p < 0.001$.

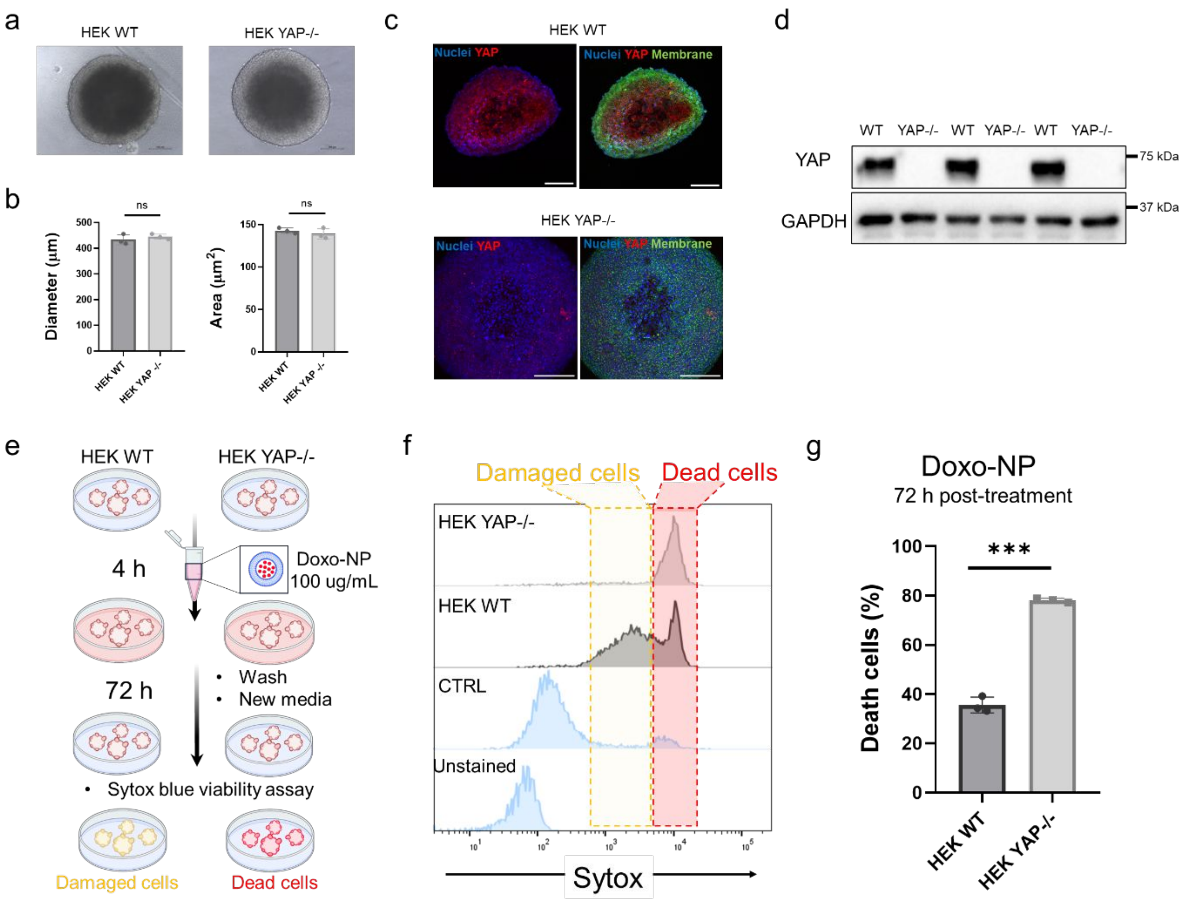

Figure S21. a) Representative brightfield images of HEK WT and HEK YAP -/- spheroids seeded onto 96-well round (U) bottom plates 3 days after seeding. b) Spheroids diameter (left) and area (right) measure after three days of culture. n = 3. Statistical analysis was done using unpaired t-test; ns, nonsignificant. c) Z-projection images of WT (top) and YAP -/- HEK (bottom) spheroids after 3 days of culture. Cells are stained with YAP (AF568, red), WGA-488 (green) and DAPI (blue). Scale bar: 200 µm. d) Western blot showing the YAP in WT and YAP-/- HEK 293T cells. GAPDH was used for protein loading normalization e) Outline of the procedure for treating WT and YAP-/- cells with Doxo-NP. After three days in culture, spheroids were treated with 100 µg/mL of NPs for 4 hours. Following washes and media replacement, the spheroids were allowed to grow for an additional 72 hours before performing the Sytox dead cell assay. f) Histogram of the sytox dead cells assay for WT and YAP -/- HEK cells, 72 hours after treatment with Doxo-NPs. Unstained cells and control cells treated with sytox (CTRL) are presented in light blue. The population of "damaged cells" is highlighted with orange dashed line box and the population of "dead cells" with red dashed line box. g) Bar plot of the percentage of dead cells for WT (dark grey) and YAP-/- (light grey) HEK cells, 72 hours after treatment with Doxo-NPs. Uptake ratio of PS100 in HEK WT (dark grey) and YAP -/- (light grey) after 4-hours of incubation with the nanoparticles. Statistical analysis was performed by unpaired t-test. n = 3; ***p < 0.001.

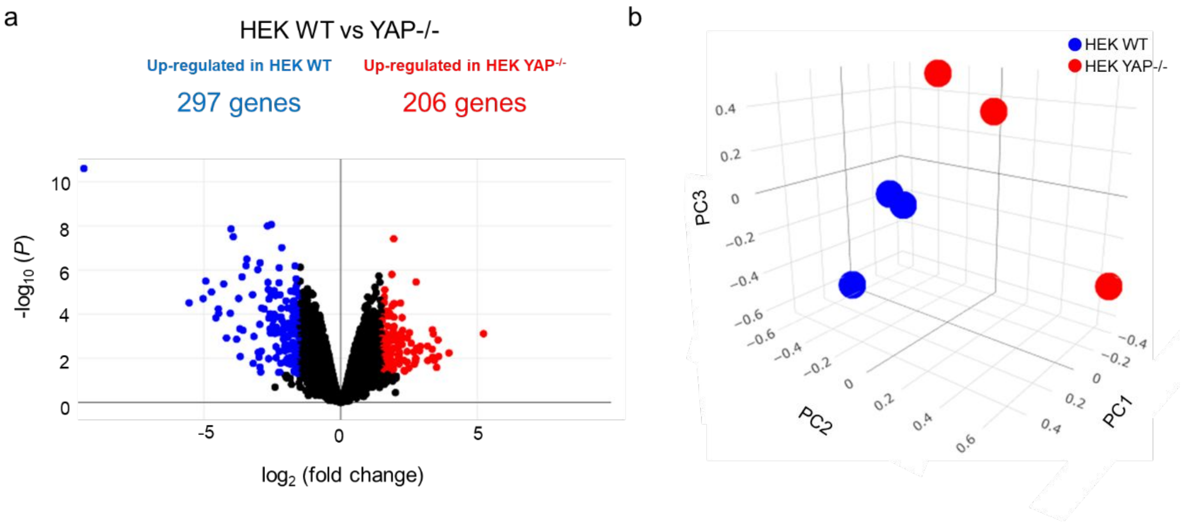

Figure S22. (a) Volcano plot showing differential gene expression in WT *vs* YAP −/− HEK 293T cells. Red markers indicate significantly upregulated genes and blue markers indicate downregulated genes; $N = 3$ (P adj < 0.05, log2FC > |1|). (b) 3D principal component (PC) analysis of RNA-seq in WT and YAP −/− HEK 293T cells. Red dots represent a sample of YAP −/− cells, whereas blue dots represent a sample of WT cells. $n = 4$ (P adj < 0.05, log2FC > |1|). The analysis was performed *via* Biojupies.[S10]

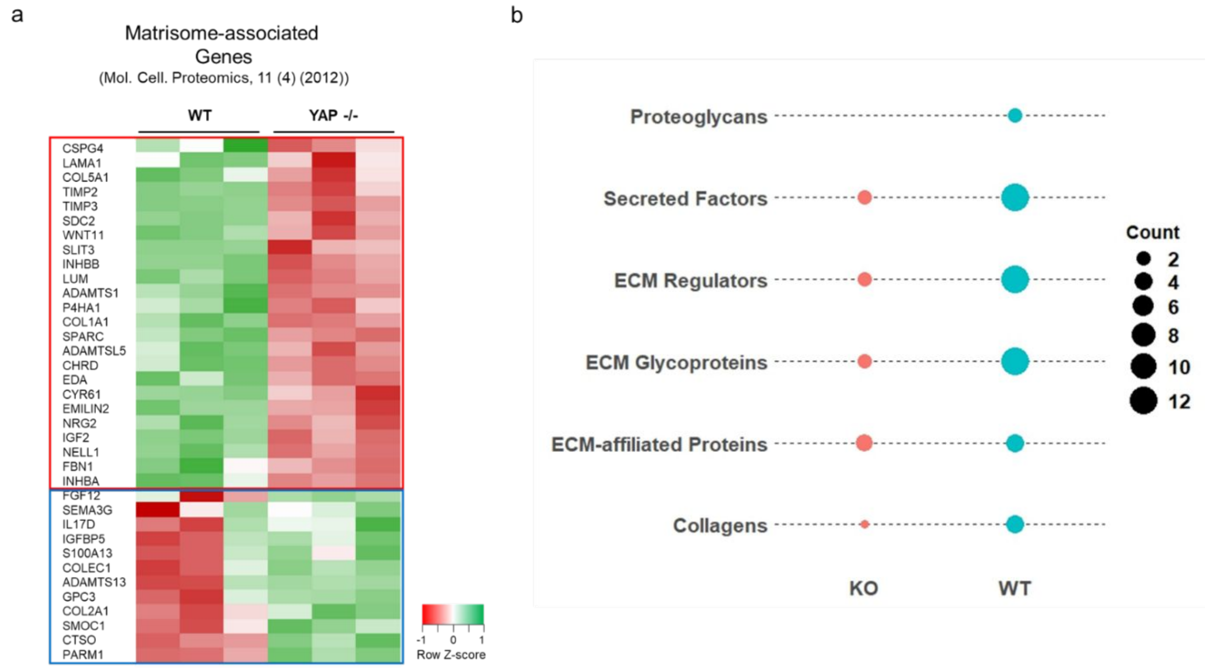

Figure S23. a) Heatmap of the relative expression of genes belonging to the human matrisome and differentially expressed between HEK WT and HEK YAP -/- (P adj < 0.05, $log_2Fc > |1|$). Red box highlights the genes upregulated in HEK WT cells, while blue box those upregulated in HEK YAP -/-. (b) Dot plot showing the expression of classes of genes belonging to the human matrisome and differentially regulated between HEK WT and YAP -/-.

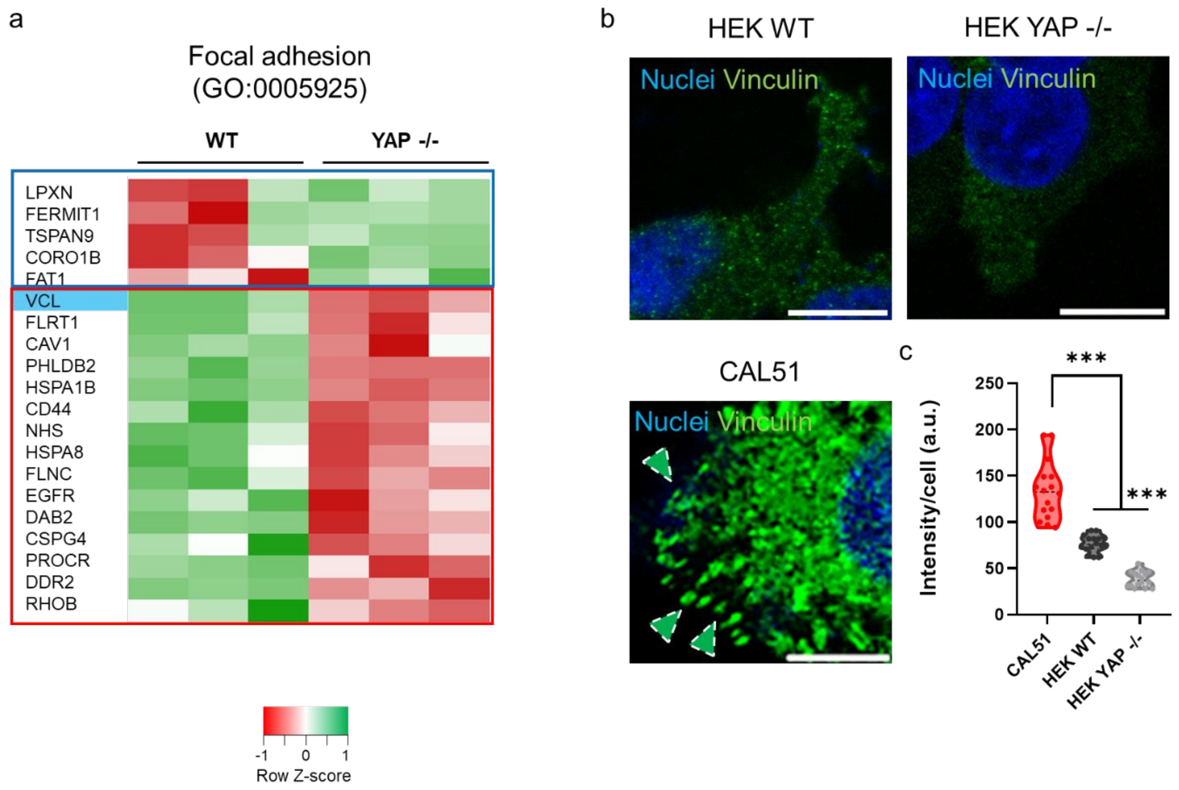

Figure S24. a) Heatmap of the relative expression of genes belonging to focal adhesion (GO:0005925) and differentially expressed between HEK WT and HEK YAP -/- (P adj < 0.05, $\log_2 Fc > |1|$). Red box highlights the genes upregulated in HEK WT cells, while blue box those upregulated in HEK YAP -/-. Vinculin (VCL) is highlighted in blue and its expression in HEK WT and YAP -/- further investigated. (b) Representative confocal images showing vinculin organization in CAL51, HEK WT and HEK YAP -/-. Cells are stained with DAPI (blue) and vinculin (AF488, green). Focal adhesion assembly in CAL51 cells is indicated with green-white dashed arrows, as shown in our previous work.[S1] Scale bar is 10 µm. (c) Violin plot of the intensity/cell of vinculin for HEK WT (dark grey) and YAP -/- (light grey) cells compared to that of CAL51 (red), characterized by intrinsically high vinculin expression and focal adhesion organization.[S2] Statistical analysis was performed *via* one-way ANOVA followed by Tukey's multiple comparison test. n > 10; ***p < 0.001.

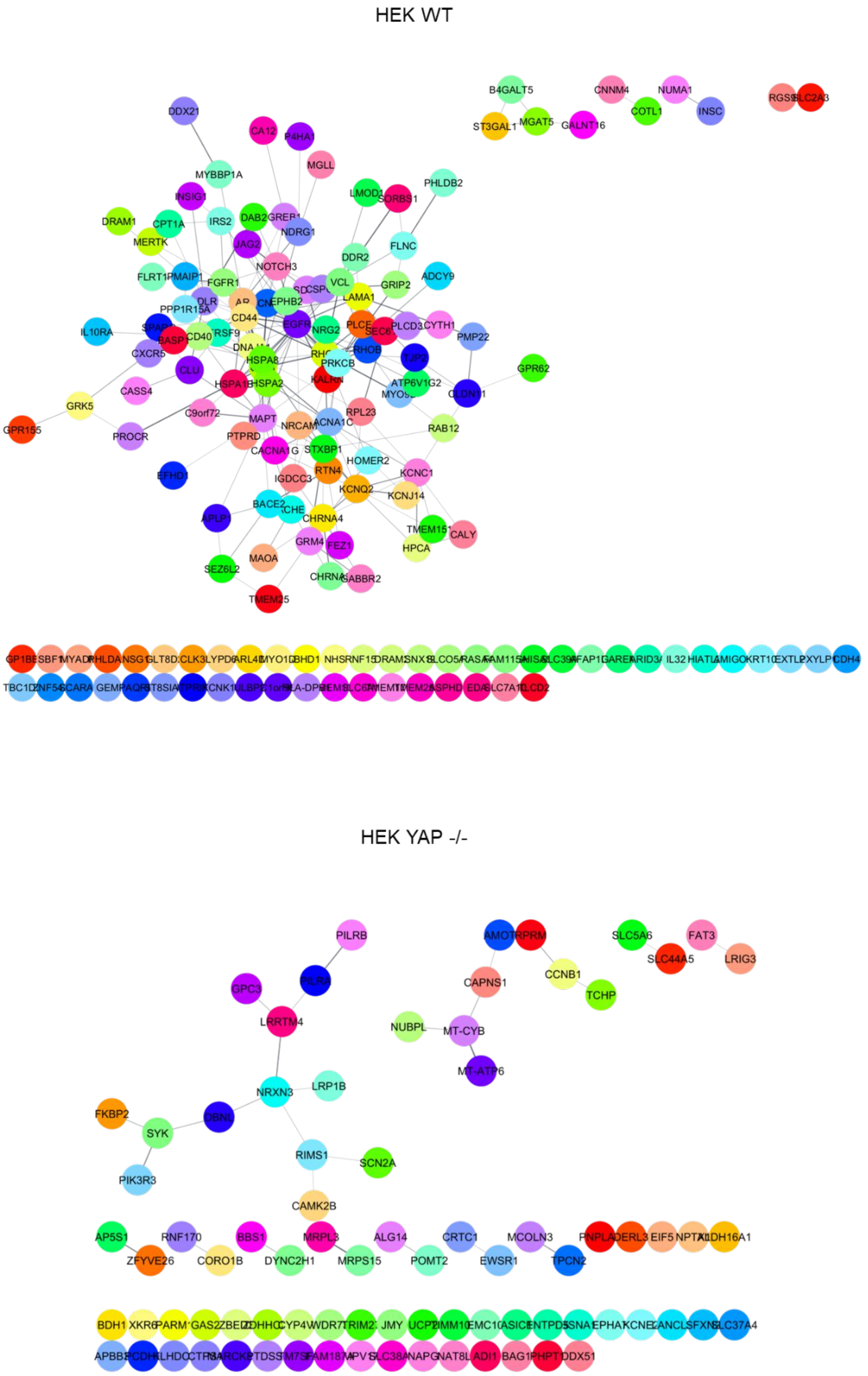

Figure S25. STRING PPI network of the differently expressed proteins involved in membrane organization (GO0016020) in WT and YAP −/− HEK cells obtained from Cytoscape (P adj < 0.05, log2FC > |1|, confidence cutoff 0.4).

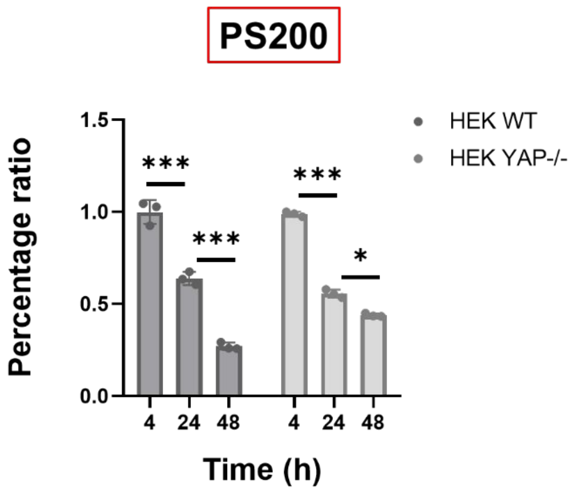

Figure S26. Comparison of the binding ratio of the HEK WT (dark grey) and YAP-/- (light grey) cells incubated with PS200 for 4 hours, followed by 24- and 48 hours culture in fresh media. Statistical analysis was performed using two-way ANOVA followed by Sidak's multiple comparison test. n= 3; ***$p < 0.001$; *$p < 0.05$.

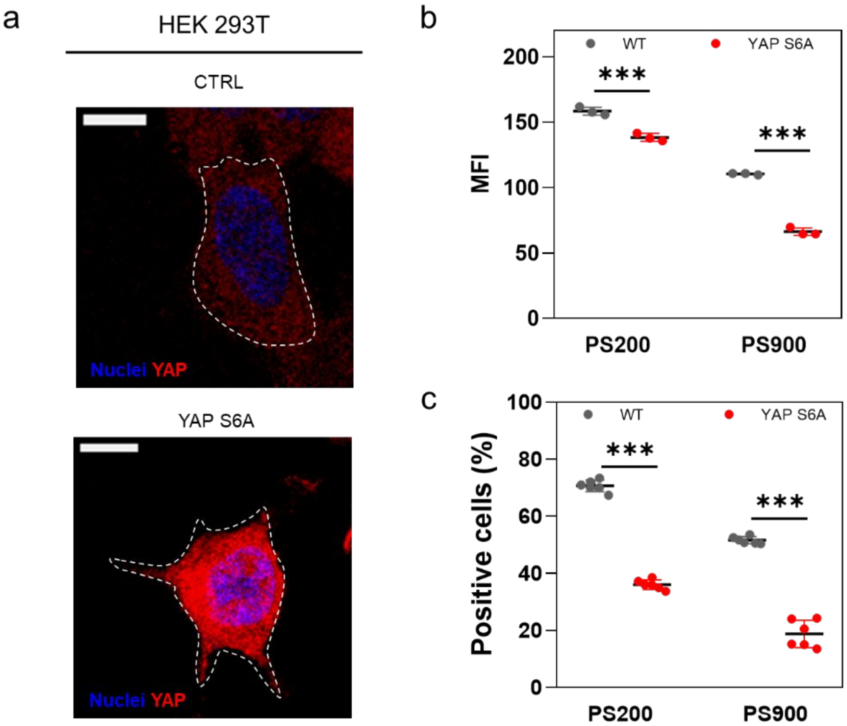

Figure S27. (a) Confocal images of WT HEK 293T cells (CTRL) and YAPS6A-transfected HEK 293T cells. Cells are stained for YAP (Alex Fluor 555, red) and DAPI (blue). Scale bars: 10 µm. (b) Median fluorescence intensity (MFI) of uptake of PS200 and PS900 in WT or YAPS6A cells following incubation for 4 h. Statistical analysis was performed using two-way ANOVA followed by Sidak's multiple comparisons test; $n = 6$; ***$p < 0.001$. (c) Uptake of PS200 and PS900 in HEK 293T WT or YAPS6A cells following incubation for 4 h. Statistical analysis was performed using two-way ANOVA followed by Sidak's multiple comparisons test; $n = 6$; ***$p < 0.001$.

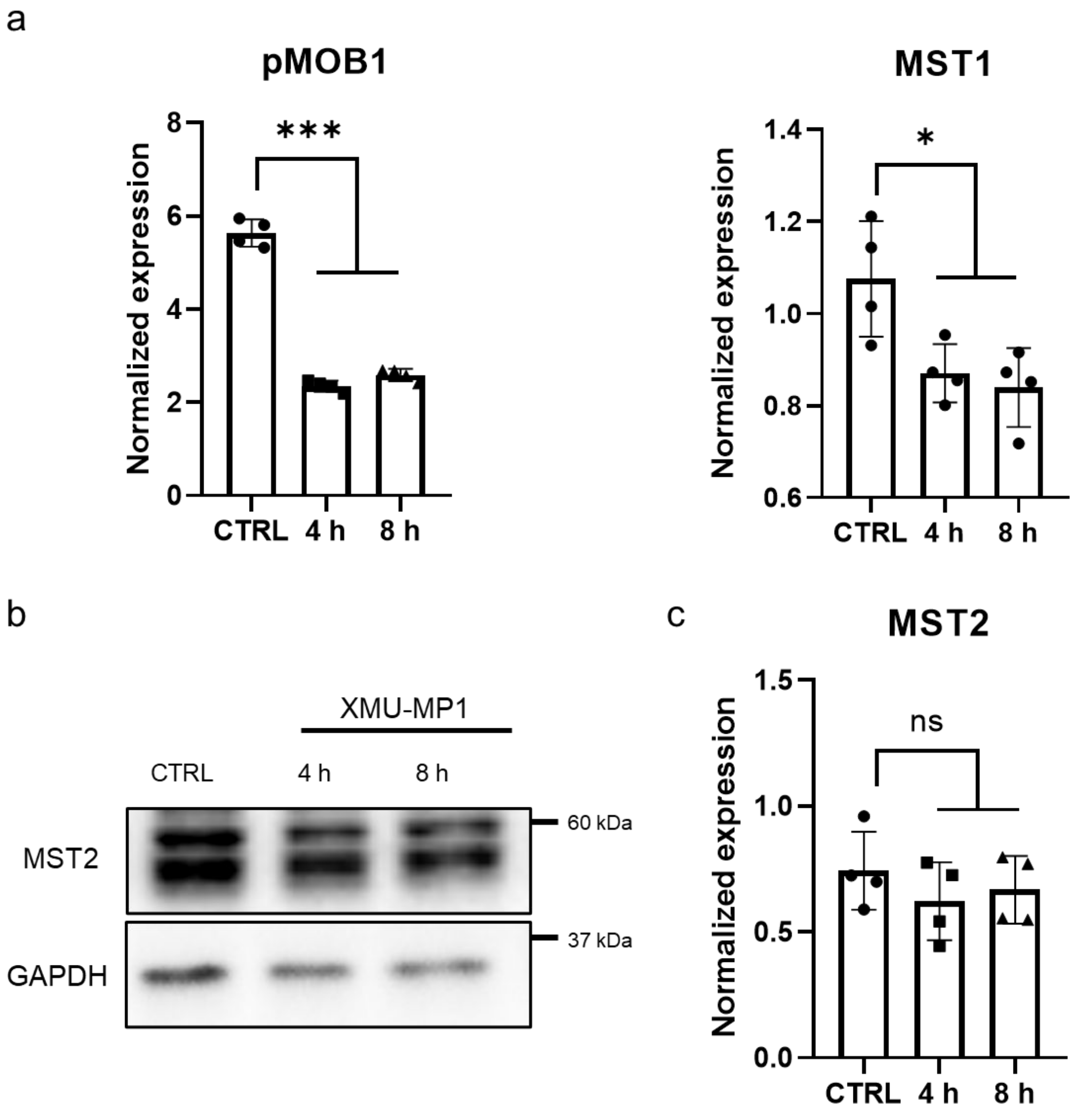

Figure S28. (a) Normalized expression of MST1 and p-MOB1 levels obtained from western blot analysis in untreated HEK 293T cells (CTRL) or HEK 293T cells treated for 4 or 8 h with 6 µM XMU-MP1 inhibitor. Statistical analysis was done *via* one-way ANOVA followed by Tukey's multiple comparison test. n = 4; ***$p < 0.001$; *$p < 0.05$. (b) Western blot showing the level of MST2 in untreated cells (CTRL) and HEK 293T treated for 4 or 8 h with 6 µM XMU-MP1 inhibitor. GAPDH was used for protein loading normalization. (c) Normalized expression of MST2 in untreated cells (CTRL) and HEK 293T cells treated for 4 or 8 h with 6 µM XMU-MP1 inhibitor.

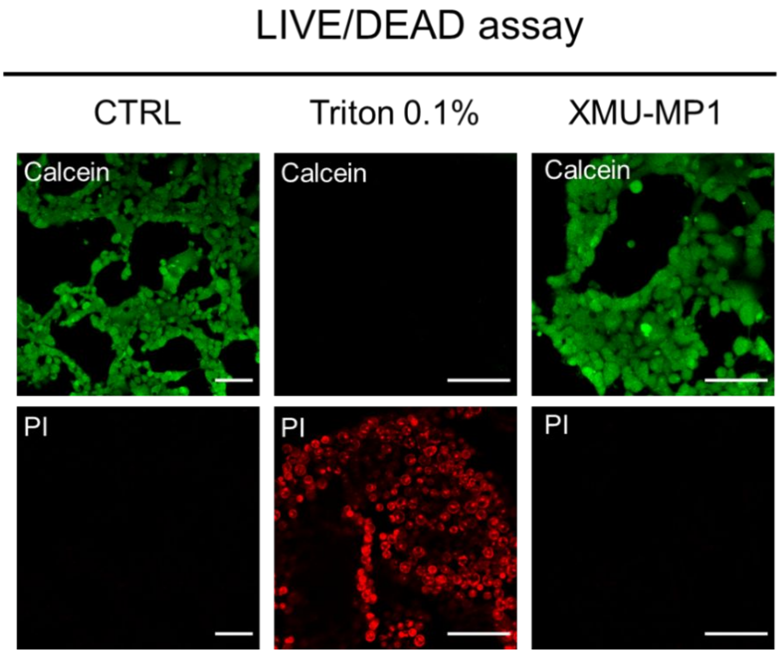

Figure S29. Live/dead assay performed on HEK 293T cells treated with XMU-MP1 for 8 h. As a control for cell death, cells were heated for 15 min with 0.1% Triton X-100 solution. Cells were excited with a 555 nm laser (propidium iodide, PI, red) and a 488 nm laser (calcein, green). Scale bars: 100 μm.

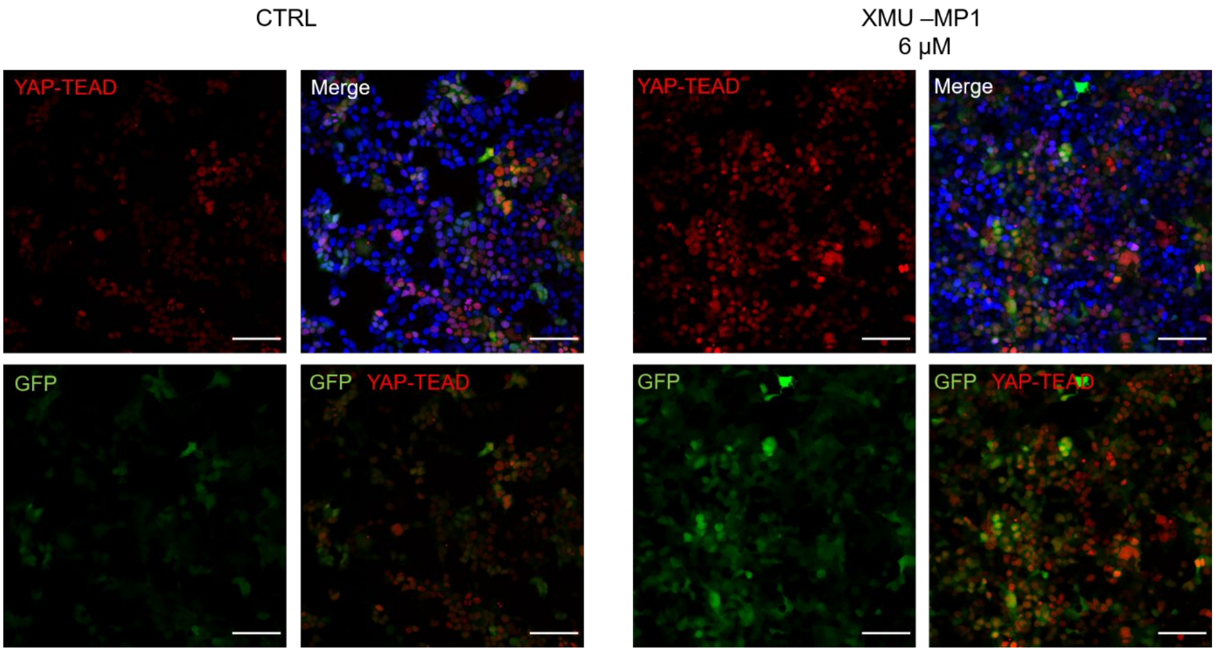

Figure S30. Representative confocal images of untreated WT HEK cells (CTRL) and HEK cells treated with 6 µM XMU-MP1 for 8 h. The green signal comes from the GFP protein coexpressed with YAP, whereas the red signal comes from the YAP-TEAD-mediated gene transcription (mCherry). Cells are stained postfixation with DAPI (Merge, blue). Scale bars: 100 µm.

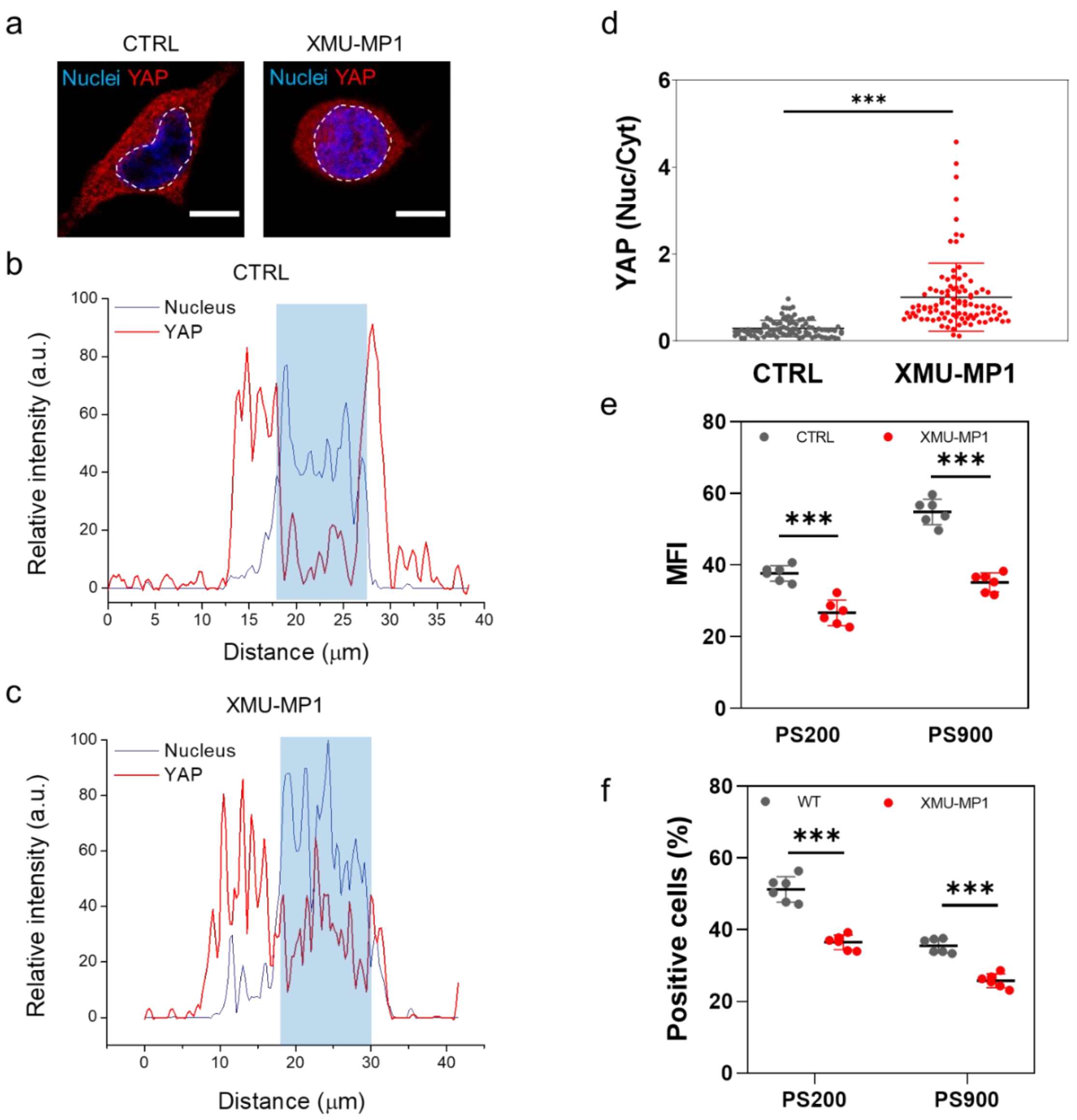

Figure S31. (a) Representative confocal images of untreated HEK 293T cells and HEK 293T cells treated with 6 μM XMU-MP1 for 8 h. The white dashed lines highlight the cell nuclei. Scale bars: 10 μm. The corresponding plot profiles of YAP signal intensity (Alex Fluor 555, red) colocalized with the nucleus (blue, DAPI) for the untreated cells (b) and XMU-MP1 treated cells (c) are also shown. (d) Dot plot representation of the YAP nuclear/cytoplasmic ratio in untreated HEK 293T cells and HEK 293T cells treated with 6 μM XMU-MP1 for 8 h. Statistical analysis was performed using unpaired *t*-test with Welch's correction; $n > 90$; $***p < 0.001$. (e) MFI of a 4-h uptake of PS200 and PS900 in untreated HEK 293T cells or HEK 293T cells treated with 6 μM XMU-MP1. Statistical analysis was performed using two-way ANOVA followed by Sidak's multiple comparisons test; $n = 6$; $***p < 0.001$. (f) Uptake of PS200 and PS900 in untreated HEK 293T cells or HEK 293T cells treated with 6 μM XMU-MP1 following incubation for 4 h. Statistical analysis was performed using two-way ANOVA followed by Sidak's multiple comparisons test; $n = 6$; $***p < 0.001$.

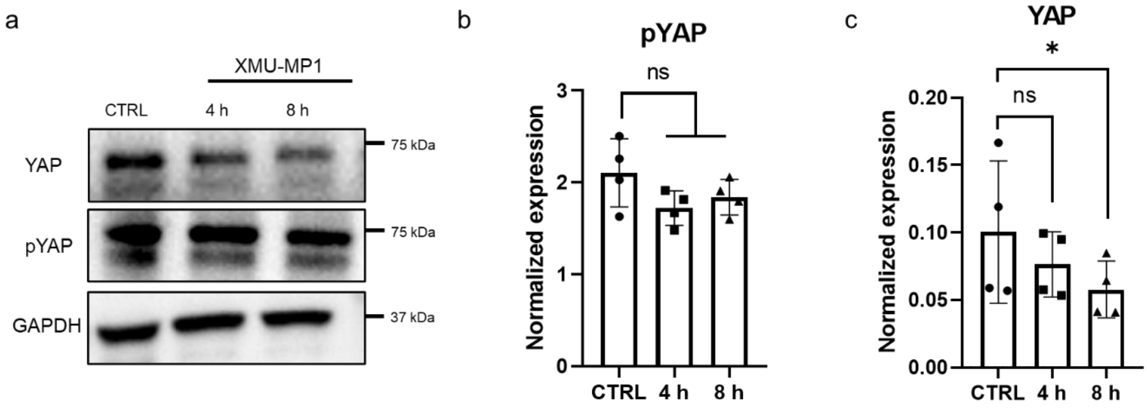

Figure S32. (a) Western blot showing the levels of YAP and p-YAP in untreated HEK 293T cells (CTRL) or HEK 293T cells treated for 4 or 8 h with 6 μM XMU-MP1 inhibitor. GAPDH was used for protein loading normalization. (b, c) Normalized expression of YAP (b) and p-YAP (c) in untreated HEK 293T cells (CTRL) and HEK 293T cells treated for 4 or 8 h with 6 μM XMU-MP1 inhibitor. (b) Statistical analysis was done using one-way ANOVA followed by Dunnett's multiple comparison test. (c) Statistical analysis was performed using two-way ANOVA followed by Dunnett's multiple comparison test. $n = 4$; $*p < 0.05$.

References

S1. Cassani, M.; Fernandes, S.; Oliver-De La Cruz, J.; Durikova, H.; Vrbsky, J.; Patočka, M.; Hegrova, V.; Klimovic, S.; Pribyl, J.; Debellis, D.; Skladal, P.; Cavalieri, F.; Caruso, F.; Forte, G., YAP Signaling Regulates the Cellular Uptake and Therapeutic Effect of Nanoparticles. *Advanced Science* **2024,** *11* (2), 2302965.
S2. Nardone, G.; Oliver-De La Cruz, J.; Vrbsky, J.; Martini, C.; Pribyl, J.; Skládal, P.; Pešl, M.; Caluori, G.; Pagliari, S.; Martino, F.; Maceckova, Z.; Hajduch, M.; Sanz-Garcia, A.; Pugno, N. M.; Stokin, G. B.; Forte, G., YAP regulates cell mechanics by controlling focal adhesion assembly. *Nature Communications* **2017,** *8* (1), 15321.
S3. Derjaguin, B. V.; Muller, V. M.; Toporov, Y. P., Effect of contact deformations on the adhesion of particles. *Journal of Colloid and Interface Science* **1975,** *53* (2), 314-326.
S4. Hermanowicz, P.; Sarna, M.; Burda, K.; Gabryś, H., AtomicJ: An open source software for analysis of force curves. **2014,** *85* (6), 063703.
S5. Yang, X.; Boehm, J. S.; Yang, X.; Salehi-Ashtiani, K.; Hao, T.; Shen, Y.; Lubonja, R.; Thomas, S. R.; Alkan, O.; Bhimdi, T.; Green, T. M.; Johannessen, C. M.; Silver, S. J.; Nguyen, C.; Murray, R. R.; Hieronymus, H.; Balcha, D.; Fan, C.; Lin, C.; Ghamsari, L.; Vidal, M.; Hahn, W. C.; Hill, D. E.; Root, D. E., A public genome-scale lentiviral expression library of human ORFs. *Nature methods* **2011,** *8* (8), 659-61.
S6. Torre, D.; Lachmann, A.; Ma'ayan, A., BioJupies: Automated Generation of Interactive Notebooks for RNA-Seq Data Analysis in the Cloud. *Cell systems* **2018,** *7* (5), 556-561.e3.
S7. Chen, E. Y.; Tan, C. M.; Kou, Y.; Duan, Q.; Wang, Z.; Meirelles, G. V.; Clark, N. R.; Ma'ayan, A., Enrichr: interactive and collaborative HTML5 gene list enrichment analysis tool. *BMC Bioinformatics* **2013,** *14* (1), 128.
S8. Kuleshov, M. V.; Jones, M. R.; Rouillard, A. D.; Fernandez, N. F.; Duan, Q.; Wang, Z.; Koplev, S.; Jenkins, S. L.; Jagodnik, K. M.; Lachmann, A.; McDermott, M. G.; Monteiro, C. D.; Gundersen, G. W.; Ma'ayan, A., Enrichr: a comprehensive gene set enrichment analysis web server 2016 update. *Nucleic acids research* **2016,** *44* (W1), W90-7.
S9. Xie, Z.; Bailey, A.; Kuleshov, M. V.; Clarke, D. J. B.; Evangelista, J. E.; Jenkins, S. L.; Lachmann, A.; Wojciechowicz, M. L.; Kropiwnicki, E.; Jagodnik, K. M.; Jeon, M.; Ma'ayan, A., Gene Set Knowledge Discovery with Enrichr. *Current protocols* **2021,** *1* (3), e90.
S10. Torre, D.; Lachmann, A.; Ma'ayan, A., BioJupies: Automated Generation of Interactive Notebooks for RNA-Seq Data Analysis in the Cloud. *Cell systems* **2018,** *7* (5), 556-561.e3.
